# No evidence of difference in mortality with amoxicillin versus co-amoxiclav for hospital treatment of community-acquired pneumonia

**DOI:** 10.1016/j.jinf.2024.106161

**Published:** 2024-06

**Authors:** Jia Wei, Aashna Uppal, Christy Nganjimi, Hermione Warr, Yasin Ibrahim, Qingze Gu, Hang Yuan, Najib M. Rahman, Nicola Jones, A. Sarah Walker, David W. Eyre

**Affiliations:** a Nuffield Department of Medicine, University of Oxford, Oxford, UK; b Department of Engineering Science, University of Oxford, Oxford, UK; c Big Data Institute, Nuffield Department of Population Health, University of Oxford, Oxford, UK; d Oxford Centre for Respiratory Medicine, Churchill Hospital, Oxford, UK; e The National Institute for Health Research Oxford Biomedical Research Centre, University of Oxford, Oxford, UK; f Department of Infectious Diseases and Microbiology, Oxford University Hospitals NHS Foundation Trust, John Radcliffe Hospital, Oxford, UK; g The National Institute for Health Research Health Protection Research Unit in Healthcare Associated Infections and Antimicrobial Resistance at the University of Oxford, Oxford, UK

**Keywords:** Community-acquired pneumonia, Amoxicillin, Co-amoxiclav, Antimicrobial resistance, 30-day mortality

## Abstract

**Objectives:**

Current guidelines recommend broad-spectrum antibiotics for high-severity community-acquired pneumonia (CAP), potentially contributing to antimicrobial resistance (AMR). We aim to compare outcomes in CAP patients treated with amoxicillin (narrow-spectrum) versus co-amoxiclav (broad-spectrum), to understand if narrow-spectrum antibiotics could be used more widely.

**Methods:**

We analysed electronic health records from adults (≥16 y) admitted to hospital with a primary diagnosis of pneumonia between 01-January-2016 and 30-September-2023 in Oxfordshire, United Kingdom. Patients receiving baseline ([−12 h,+24 h] from admission) amoxicillin or co-amoxiclav were included. The association between 30-day all-cause mortality and baseline antibiotic was examined using propensity score (PS) matching and inverse probability treatment weighting (IPTW) to address confounding by baseline characteristics and disease severity. Subgroup analyses by disease severity and sensitivity analyses with missing covariates imputed were also conducted.

**Results:**

Among 16,072 admissions with a primary diagnosis of pneumonia, 9685 received either baseline amoxicillin or co-amoxiclav. There was no evidence of a difference in 30-day mortality between patients receiving initial co-amoxiclav vs. amoxicillin (PS matching: marginal odds ratio 0.97 [0.76–1.27], p = 0.61; IPTW: 1.02 [0.78–1.33], p = 0.87). Results remained similar across stratified analyses of mild, moderate, and severe pneumonia. Results were also similar with missing data imputed. There was also no evidence of an association between 30-day mortality and use of additional macrolides or additional doxycycline.

**Conclusions:**

There was no evidence of co-amoxiclav being advantageous over amoxicillin for treatment of CAP in 30-day mortality at a population-level, regardless of disease severity. Wider use of narrow-spectrum empirical treatment of moderate/severe CAP should be considered to curb potential for AMR.

## Highlights

• We compared 30-day mortality in community-acquired pneumonia (CAP) patients treated with amoxicillin or co-amoxiclav.
• We used propensity score matching and inverse probability treatment weighting to account for confounding.
• There was no evidence that amoxicillin instead of co-amoxiclav for treatment of CAP was harmful.
• Results were consistent across CAP severity levels.
• Wider use of amoxicillin in empiric treatment of CAP should be considered.

## Introduction

Community-acquired pneumonia (CAP) is a leading cause of morbidity and mortality globally, particularly affecting older and medically vulnerable people.^1^ In the United Kingdom (UK), annual CAP incidence ranges between 22–80/10,000 persons.^2^, ^3^, ^4^, ^5^ A broad range of pathogens cause CAP, including bacteria such as *Streptococcus pneumoniae*, *Haemophilus influenzae, Staphylococcus aureus, Mycoplasma pneumoniae*, *Legionella pneumophila*, *Klebsiella pneumoniae,* and viruses such as influenza, respiratory syncytial virus (RSV), and SARS-CoV-2.^6^, ^7^, ^8^

Antibiotics and supportive care are the main treatments for bacterial CAP; most clinically treated cases do not have the causative pathogen identified,^9^, ^10^, ^11^ so most treatment is empirical. In the UK and Northern Europe, where most *S. pneumoniae* remain penicillin susceptible, narrow-spectrum beta-lactam treatment principally targeting *S. pneumoniae*, e.g. oral penicillin or ampicillin/amoxicillin, is often effective. However, concerns about other pathogens such as *K. pneumoniae* and beta-lactamase producing *H. influenzae* and *S. aureus*, which may not respond to such treatment, can lead to use of broader-spectrum beta-lactams, with macrolides/doxycycline also added to cover ‘atypical’ infections such as *Mycoplasma*.

Current guidelines recommend amoxicillin for low/moderate-severity CAP, ±a macrolide if atypical infection is suspected in moderate-severity cases, and co-amoxiclav + a macrolide for high-severity CAP.^12^, ^13^ Broader-spectrum antibiotics are recommended for severe disease, to ensure less common, but more resistant, pathogens are treated, given the greater potential for adverse outcomes. However, concern about resistance and prior community treatment with narrow-spectrum antibiotics, can lead to use of co-amoxiclav and other broad-spectrum beta-lactams in non-severe CAP presenting to hospital. Use and over-use of broad-spectrum antibiotics contributes to antimicrobial resistance (AMR),^14^ more side effects, and increased *Clostridioides difficile* infections.^15^, ^16^ Understanding when narrower-spectrum antibiotics can safely be used could help mitigate AMR development.

While population-based studies and clinical trials have examined the effectiveness of different antibiotic treatments for CAP,^6^ none have directly compared the treatment outcomes between amoxicillin and co-amoxiclav, the two most commonly used antibiotics for CAP in the UK. We used electronic healthcare records (EHR) from a large UK teaching hospital group to investigate clinical outcomes in patients with CAP treated with either amoxicillin or co-amoxiclav, to understand if narrow-spectrum antibiotics could be safely used more widely.

## Methods

### Patients and setting

De-identified electronic patient record data were obtained from Oxford University Hospitals (OUH) NHS Foundation Trust using the Infections in Oxfordshire Research Database (IORD) which has Research Ethics Committee, Health Research Authority and Confidentiality Advisory Group approvals (19/SC/0403,19/CAG/0144). OUH consists of four teaching hospitals in Oxfordshire, UK, with a total of ∼1100 beds, serving a population of ∼755,000 people and providing specialist services to the surrounding region. Out-of-hospital mortality is determined by regular updates from the national information system recording all UK deaths.

We included all adult patients (≥16 years old) admitted to OUH between 01-January-2016 and 30-September-2023 with a primary diagnosis code of pneumonia (ICD-10 J13-J18). Within each hospital admission, diagnosis codes are assigned separately by episodes (periods of care under a specific specialty/consultant). We focused on CAP and included patients with a pneumonia primary diagnosis code in the first episode per admission, i.e., only considering patients where pneumonia was the reason for hospital admission. We excluded patients with a SARS-CoV-2 infection secondary diagnosis codes (U07.1/U07.2) and patients admitted 01-February-2020 to 31-May-2020 (i.e., prior to widespread SARS-CoV-2 testing) to avoid including patients with COVID-19 pneumonia. Linked microbiology and radiology data were used to assess the performance of coding data for identifying pneumonia (Supplementary Methods).

### Outcome, exposures, and covariates

We investigated the effect of baseline antibiotics received on 30-day all-cause mortality (in-hospital or post-discharge) from the admission date. All antibiotics prescribed in hospital, intravenous/oral and inpatient/post-discharge, received within [−12,+24 h] of admission were considered as baseline antibiotics. As main exposures, we compared patients who received baseline amoxicillin versus baseline co-amoxiclav. For patients who received amoxicillin or co-amoxiclav in combination with other antibiotics, we included as separate binary variables whether patients received additional baseline macrolides (clarithromycin/azithromycin/erythromycin), doxycycline, or gentamicin. Although gentamicin would not provide effective treatment for pneumonia, adding gentamicin to a beta-lactam was part of hospital guidelines for managing sepsis, so provided some adjustment for clinician assessment of disease severity. We excluded patients who did not receive either amoxicillin or co-amoxiclav at baseline, and those who received a mixture of amoxicillin and co-amoxiclav at baseline. We also excluded patients who received any antibiotics other than those listed above in the baseline period (Table S1; fluroquinolone use was uncommon (<3 % episodes)).

To adjust as much as possible for disease severity at presentation and its impact on treatment choice, we considered as baseline covariates: age, sex, ethnicity, index of multiple deprivation (IMD) percentile, admission specialty, admission hour of day (0–8 h, 8–11 h, 11–15 h, 15–24 h^17^), admission day of the week, calendar time, hospital admission in the past year (binary), hospital length of stay in the past year, Charlson co-morbidity score, hospital frailty risk score,^18^ additional specific co-morbidities (recent urinary tract infection (UTI), immunosuppression, palliation, autoimmune diseases), admission vital signs, laboratory tests, and pneumonia risk prediction scores (CURB-65, PSI/PORT, Smart COP) (see Supplementary Methods). Smoking status was not available.

Admission vital signs were the closest measurements to the date/time of admission obtained in the [−24,+12 h] baseline window, to include observations in the Emergency Department before admission. We included the following vital signs (continuous unless otherwise stated): heart rate, respiratory rate, systolic and diastolic blood pressure, temperature, oxygen saturations, use of oxygen (binary), litres of oxygen delivered > 1 litre/minute, and AVPU status (Alert, response to Voice, response to Pain, Unresponsive). Missing AVPU measurements were completed using the Glasgow Coma Scale (GCS) when available, classified as: 15: Alert, 9–14: Voice, 4–8: Pain, 3: Unresponsive. We assumed patients with missing AVPU and GCS were ‘Alert’. National Early Warning Score 2 (NEWS2) values were calculated from vital signs and included in models.^19^

Admission laboratory tests were defined similarly, including haemoglobin, mean cell volume (MCV), neutrophils, lymphocytes, monocytes, eosinophils, platelets, sodium, potassium, urea, bilirubin, alanine transferase (ALT), alkaline phosphatase (ALP), albumin, C-reactive protein (CRP), prothrombin time (PT), activated partial thromboplastin time (APTT), lactate (venous or arterial), and pH (dichotomised into <7.25 and ≥7.25 to reflect acidosis). Creatinine and white cell count were strongly correlated with urea and neutrophils/monocytes respectively (Pearson correlation coefficient>0.8), and hence were excluded from models.

### Statistical methods

In contrast to randomised treatment assignment in a trial, in observational/EHR studies multiple factors influence treatment choice, e.g. patient comorbidities or CAP severity, leading to treatments given for more severe diseases potentially being associated with worse outcomes. Therefore, to estimate the effect of baseline amoxicillin vs. co-amoxiclav on 30-day all-cause mortality following CAP, we used two causal methods designed to account for this.^20^ Provided all factors influencing treatment assignment are modelled, they can produce estimates of treatment effects comparable to estimates from experimental trials. We assessed consistency between the two methods, and also compared with a standard multivariable logistic regression model (Supplementary Methods). To emulate a target trial, i.e. to provide a causal estimate of the effect of initiating antibiotics with co-amoxiclav (treatment) vs. amoxicillin (control), we used propensity score (PS) matching and inverse probability treatment weighting (IPTW), to estimate the average treatment effect in the population,^21^ i.e. the average effect at the population level if all patients received co-amoxiclav vs. all received amoxicillin. Propensity scores were calculated using multivariable logistic regression with baseline antibiotic as the outcome, including all baseline covariates and allowing non-linear and interaction terms (Supplementary Methods). PS model covariates were based on subject-matter knowledge of likely associations with 30-day mortality.

For PS matching, we used optimal full matching, matching patients in either treatment or control groups to ≥ 1 patient in the alternative group based on PS^22^ to minimise overall differences between groups without discarding any patients. IPTW used sampling weights to create a quasi-randomised synthetic sample, truncating extreme weights to optimise covariate balance targeting standardised mean differences (SMD) < 0.1.^23^ Following matching or weighting, treatment effects on 30-day mortality were estimated using logistic regression with cluster-robust standard errors, calculating marginal odds ratios through G-computation.^24^ Sensitivity analyses used PS stratification (by quintiles^21^) and doubly-robust estimation, which increases robustness to model mis-specification and unbiasedly estimates treatment effects even when only one of the PS or outcome models is correctly specified.^25^ Subgroup analyses stratified by baseline CURB-65 pneumonia severity score (see Supplementary Methods), classifying scores 0–1 as mild, 2 as moderate, and 3–5 as moderate/severe.^13^

Primary analyses used complete cases. Sensitivity analyses used multiple imputation with chained equations (MICE) with classification and regression trees.^26^ PS matching and IPTW were then applied within each imputed dataset,^27^ with pooled marginal odds ratios calculated across 25 imputed datasets using Rubin’s rules.

## Results

**Fig. 1:**
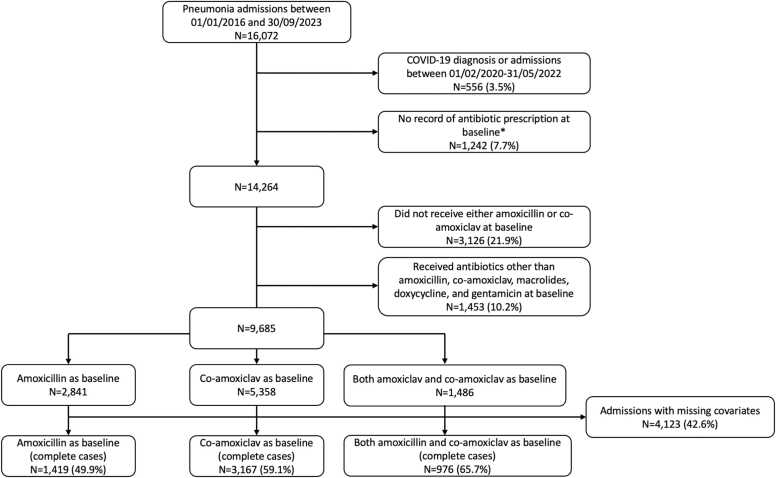
Study inclusion and exclusion flow chart. *including some patients admitted directly to the Intensive Care Unit from where prescribing data was not available.

Between 01-January-2016 and 30-September-2023, 16,072 admissions had a primary diagnostic code consistent with pneumonia. Excluding patients with SARS-CoV-2 infection or receiving baseline antibiotics other than amoxicillin, co-amoxiclav, macrolides, doxycycline, and gentamicin, 9685 admissions were included in analysis (Fig. 1). Median (interquartile range [IQR]) age was 79.0 (66.5–87.3) years; 4614 (47.6 %) patients were female. Median (IQR) Charlson score was 1 (1–2) and frailty score was 7.3 (2.0–16.3). 804 patients (8.3 %) had immunosuppression, 1202 (12.4 %) had autoimmune disease, and 796 (8.2 %) were coded as receiving palliative care within the preceding year. 752 (7.8 %) had a hospital diagnostic UTI code within the last year. 4730 (48.8%) patients had been admitted to OUH in the past year, with a median (IQR) total length of stay of 4.7 (0.6–24.9) days (Table 1; Table S2 for details of admission vital signs and laboratory measurements).

**Table 1:** Baseline characteristics by initial antibiotics received.

	Amoxicillin (N = 2841)	Co-amoxiclav (N = 5358)	Both (N = 1486)	Total (N = 9685)	p-value
**30-day mortality**					< 0.001
**No**	2599 (91.5 %)	4335 (80.9 %)	1298 (87.3 %)	8232 (85.0 %)	
**Yes**	242 (8.5 %)	1023 (19.1 %)	188 (12.7 %)	1453 (15.0 %)	
**Age, years**					< 0.001
**Median (Q1, Q3)**	77.6 (62.9, 86.9)	79.5 (67.8, 87.5)	79.7 (68.4, 87.5)	79.0 (66.5, 87.3)	
**Sex**					< 0.001
**Female**	1446 (50.9 %)	2491 (46.5 %)	677 (45.6 %)	4614 (47.6 %)	
**Male**	1395 (49.1 %)	2867 (53.5 %)	809 (54.4 %)	5071 (52.4 %)	
**Ethnicity**					0.85
**White**	109 (4.5 %)	203 (4.4 %)	52 (4.1 %)	364 (4.4 %)	
**Non-White**	2296 (95.5 %)	4380 (95.6 %)	1208 (95.9 %)	7884 (95.6 %)	
**Missing, N**	436	775	226	1437	
**Deprivation percentile**					0.55
**Median (Q1, Q3)**	9.7 (6.1, 15.7)	10.1 (6.2, 15.7)	10.2 (6.1, 15.9)	10.0 (6.1, 15.7)	
**Missing, N**	32	61	12	105	
**Additional gentamicin**					< 0.001
**No**	2794 (98.3 %)	4640 (86.6 %)	1350 (90.8 %)	8784 (90.7 %)	
**Yes**	47 (1.7 %)	718 (13.4 %)	136 (9.2 %)	901 (9.3 %)	
**Additional macrolide**					< 0.001
**No**	2248 (79.1 %)	3607 (67.3 %)	911 (61.3 %)	6766 (69.9 %)	
**Yes**	593 (20.9 %)	1751 (32.7 %)	575 (38.7 %)	2919 (30.1 %)	
**Additional doxycycline**					< 0.001
**No**	2023 (71.2 %)	4867 (90.8 %)	914 (61.5 %)	7804 (80.6 %)	
**Yes**	818 (28.8 %)	491 (9.2 %)	572 (38.5 %)	1881 (19.4 %)	
**Consultant specialty**					< 0.001
**Acute medicine**	1219 (42.9 %)	2380 (44.4 %)	611 (41.1 %)	4210 (43.5 %)	
**Emergency Medicine**	219 (7.7 %)	183 (3.4 %)	56 (3.8 %)	458 (4.7 %)	
**Gerontology**	751 (26.4 %)	1430 (26.7 %)	401 (27.0 %)	2582 (26.7 %)	
**Infectious disease**	310 (10.9 %)	539 (10.1 %)	210 (14.1 %)	1059 (10.9 %)	
**Other**	342 (12.0 %)	826 (15.4 %)	208 (14.0 %)	1376 (14.2 %)	
**Charlson comorbidity score**					< 0.001
**Median (Q1, Q3)**	1 (0, 2)	2 (1, 3)	1 (1, 2)	1 (1, 2)	
**Frailty score**					< 0.001
**Median (Q1, Q3)**	4.6 (0.7, 12.9)	8.5 (2.9, 17.7)	7.7 (2.1, 16.7)	7.3 (2.0, 16.3)	
**Admitted to hospital in previous year**					< 0.001
**No**	1604 (56.5 %)	2564 (47.9 %)	787 (53.0 %)	4955 (51.2 %)	
**Yes**	1237 (43.5 %)	2794 (52.1 %)	699 (47.0 %)	4730 (48.8 %)	
**Length of hospital stay in previous year (days)**				< 0.001	
**Median (Q1, Q3)**	3.0 (0.4, 16.4)	6.1 (0.8, 29.8)	3.8 (0.5, 20.5)	4.7 (0.6, 24. 9)	
**Had hospital UTI code in previous year**					< 0.001
**No**	2692 (94.8 %)	4869 (90.9 %)	1372 (92.3 %)	8933 (92.2 %)	
**Yes**	149 (5.2 %)	489 (9.1 %)	114 (7.7 %)	752 (7.8 %)	
**Had palliative care code in previous year**					< 0.001
**No**	2708 (95.3 %)	4795 (89.5 %)	1386 (93.3 %)	8889 (91.8 %)	
**Yes**	133 (4.7 %)	563 (10.5 %)	100 (6.7 %)	796 (8.2 %)	
**Had immunosuppression code in previous year**				< 0.001	
**No**	2670 (94.0 %)	4819 (89.9 %)	1392 (93.7 %)	8881 (91.7 %)	
**Yes**	171 (6.0 %)	539 (10.1 %)	94 (6.3 %)	804 (8.3 %)	
**Had autoimmune disease code in previous year**				0.09	
**No**	2514 (88.5 %)	4658 (86.9 %)	1311 (88.2 %)	8483 (87.6 %)	
**Yes**	327 (11.5 %)	700 (13.1 %)	175 (11.8 %)	1202 (12.4 %)	
**Severity by CURB-65 score**					< 0.001
**Mild (0-1)**	1374 (52.7 %)	1922 (38.3 %)	585 (40.7 %)	3881 (42.8 %)	
**Moderate (2)**	857 (32.9 %)	1788 (35.7 %)	528 (36.7 %)	3173 (35.0 %)	
**Severe (3-5)**	377 (14.5 %)	1305 (26.0 %)	324 (22.6 %)	2006 (22.2 %)	
**Missing, N**	233	343	49	625	

Among 9685 admissions, 5871 (60.6 %) had blood cultures performed and 298 (3.1 %) had a positive blood culture with a pneumonia-associated pathogen. 1532 (15.8 %) were tested with influenza/RSV PCR, 80 (0.8 %) had influenza and 55 (0.6 %) RSV detected. 373 (3.9 %) patients (predominantly immunosuppressed) were tested with a multiplex respiratory PCR, 7 (<0.1 % overall) had *Mycoplasma* detected. 1113 (11.5 %) received a legionella urinary antigen test, only 2 had a positive result*.* In patients with positive blood cultures, the most common pathogen identified was *S. pneumoniae* (195 admissions, 2.0 % of all admissions)*,* followed by *S. aureus* (40, 0.4 %), *K. pneumoniae* (29, 0.3 %), *Pseudomonas aeruginosa* (23, 0.2 %), and *H. influenzae* (10, 0.1 %). 183/187 (97.9 %) *S. pneumoniae* isolates were susceptible to penicillin. Penicillin susceptibility results for *S. aureus* were not routinely reported for blood culture isolates (historically <5 % of isolates were susceptible). 7/10 (70 %) of *H. influenzae* were ampicillin resistant.

9038 (93.3 %) had ≥ 1 chest X-ray (CXR) and/or CT scan during hospital admission, of which 7082 (78.4 %) were reported as showing evidence of pneumonia.

### Antibiotics received

At baseline ([−12,+24 h] of admission), 2841 (29.3 %) patients received amoxicillin but not co-amoxiclav, with a median (IQR) duration of all antibiotic treatment, including switching agents/route, of 5.4 (5.0–7.3) days (5.0 (4.7–6.5) days on amoxicillin). 1628 (57.3 %) received oral, 664 (23.4 %) intravenous, and 546 (19.2 %) both oral and intravenous amoxicillin (3 through other routes). 5358 (55.3 %) patients received baseline co-amoxiclav, with a median 6.8 (5.1–10.0) total days of antibiotics (5.9 (4.7–7.9) days co-amoxiclav). 575 (10.7 %), 3568 (66.6 %), and 1212 (22.6 %) received oral, intravenous, and both oral and intravenous co-amoxiclav, respectively (3 through other routes). 1486 (15.3 %) received both amoxicillin and co-amoxiclav at baseline with a median 6.2 (5.2–8.8) total days of antibiotics. Among those receiving amoxicillin initially, 469 (16.5%) later switched to or received additional co-amoxiclav after the baseline period, after a median (IQR) 4.1 (1.7–5.3) days. Conversely, among those receiving co-amoxiclav initially, 499 (9.3 %) later de-escalated to amoxicillin, after a median (IQR) 2.4 (1.3–5.2) days. At baseline, 2919 (30.1 %) received additional macrolide antibiotics, 1881 (19.4 %) additional doxycycline, and 901 (9.3 %) additional gentamicin (Table 1).

Multiple factors were independently associated with baseline antibiotic use, co-amoxiclav was more likely to be prescribed in males, admissions to acute medicine, patients with immunosuppression, higher respiratory rate, higher heart rate, higher oxygen flowrate, with more abnormal laboratory blood measurements, higher lactate, and pH < 7.25 (Table 2, Figs. S1, S2). The probability of co-amoxiclav prescription also varied by year, peaking in 2016, then gradually declining before increasing in 2019, followed by a decline in late 2021 (Fig. S1).

**Table 2:** Associations (odds ratios (ORs) and 95 % confidence intervals (95 %CIs)) between baseline characteristics and initial receipt of co-amoxiclav vs. amoxicillin. The 95 % CIs are calculated as estimates ± 1.96 × standard error of the estimates. Non-linear effects are shown in Figs. S1, S2. UTI: urinary tract infection; ALP: alkaline phosphatase; ALT: alanine transferase.

	OR (Co-amoxiclav vs amoxicillin)	Lower CI	Upper CI	p-value
**Age (per 10 years older)**	0.91	0.83	0.99	**0.03**
**Sex (Male vs Female)**	1.19	1.02	1.40	**0.03**
**Ethnicity (Non-white vs White)**	1.34	0.88	2.08	0.18
**IMD score (per 10 higher)**	1.08	0.99	1.17	0.07
**Consultant specialty (Emergency Medicine vs Acute Medicine)**	0.56	0.39	0.81	**0.002**
**Consultant specialty (Gerontology vs Acute Medicine)**	0.72	0.61	0.86	**< 0.001**
**Consultant specialty (Infectious disease vs Acute Medicine)**	0.65	0.52	0.81	**< 0.001**
**Consultant specialty (Other vs Acute Medicine)**	0.89	0.72	1.11	0.31
**Palliative care (Yes vs No)**	1.06	0.79	1.43	0.70
**UTI in previous year (Yes vs No)**	1.15	0.86	1.55	0.34
**Immunosuppression (Yes vs No)**	1.31	0.97	1.78	0.08
**Autoimmune diseases (Yes vs No)**	0.99	0.8	1.23	0.93
**Charlson score (per 1 higher)**	0.94	0.87	1.00	0.07
**Length of hospital stay in previous year (per 10 days longer)**	0.99	0.96	1.03	0.67
**Hospital admission in previous year (Yes vs No)**	1.04	0.87	1.23	0.68
**Admission hour (0-8 vs 11-15)**	1.56	1.24	1.96	**< 0.001**
**Admission hour (8-11 vs 11-15)**	1.20	0.93	1.57	0.17
**Admission hour (15-24 vs 11-15)**	1.09	0.91	1.31	0.34
**Admission date (Monday vs Wednesday)**	1.26	0.97	1.65	0.09
**Admission date (Tuesday vs Wednesday)**	0.98	0.76	1.26	0.87
**Admission date (Thursday vs Wednesday)**	1.02	0.79	1.32	0.87
**Admission date (Friday vs Wednesday)**	1.19	0.91	1.54	0.19
**Admission date (Saturday vs Wednesday)**	1.16	0.90	1.50	0.25
**Admission date (Sunday vs Wednesday)**	1.06	0.81	1.38	0.66
**Respiratory rate**	1.03	1.01	1.06	**0.01**
**Oxygen saturation (per 5 % higher)**	0.92	0.79	1.08	0.32
**Use of oxygen supplement (Yes vs No)**	1.12	0.82	1.53	0.47
**Oxygen flow rate**	1.09	1.04	1.14	**< 0.001**
**NEWS2 score**	0.96	0.89	1.04	0.34
**Albumin**	0.97	0.95	0.99	**0.01**
**ALP (per 50 higher)**	0.99	0.92	1.08	0.85
**ALT (per 10 higher)**	1.04	1.01	1.08	**0.03**
**Bilirubin (per 10 higher)**	0.93	0.83	1.03	0.16
**Haemoglobin (per 10 higher)**	0.99	0.94	1.04	0.61
**Neutrophils**	1.05	1.03	1.07	**< 0.001**
**Urea**	0.99	0.97	1.01	0.49
**Lactate**	1.16	1.06	1.28	**0.002**
**pH<7.25 (Yes vs No)**	2.33	1.13	5.35	**0.03**

### Unadjusted association between baseline antibiotics and 30-day mortality

Unadjusted 30-day all-cause mortality was highest in patients receiving baseline co-amoxiclav 1023/5358 (19.1 %), followed by both co-amoxiclav and amoxicillin 188/1486 (12.7 %), and lowest in those receiving baseline amoxicillin only 242/2841 (8.5 %) (Table 1). These variations likely reflect differences in prescribing practice based on disease severity and underlying comorbidities, hence a causal inference-based approach was used to account for this.

### Causal estimates of the effect of baseline antibiotic on 30-day mortality

Of 5562 (57.4 %) CAP episodes with data available for all covariates, 4586 received a single antibiotic (co-amoxiclav or amoxicillin) and were included in the main analysis (also see separate sensitivity analysis below including all cases by imputing missing data.) Using PS matching to estimate the causal association between baseline antibiotic choice and mortality, 1419 patients receiving amoxicillin and 3167 patients receiving co-amoxiclav were matched to ≥ 1 patient in the alternate group, providing 4586 original and 4586 matched patients, with balance achieved between groups across most covariates (Table S3; Fig. S3 shows the distribution of estimated PS between groups).

After matching, there was no evidence of a difference in 30-day mortality between patients receiving initial co-amoxiclav vs. amoxicillin (marginal odds ratio [OR]=0.92 [0.67–1.25], p = 0.59). Further adjustments for the small number of covariates with SMD> 0.1 to mitigate bias from residual imbalance, were consistent (marginal OR=0.95 [0.73–1.23], p = 0.67) (Table 3). Consistent findings were also observed in sensitivity analyses using PS stratification and doubly robust estimation (marginal OR=1.02 [0.89–1.16], p = 0.81; and 0.99 [0.84–1.15], p = 0.86, respectively) (Table S4A).

**Table 3:** Average treatment effects (marginal odds ratios (ORs) and 95 % confidence intervals (CIs)) of baseline co-amoxiclav vs amoxicillin on 30-day all-cause mortality. Propensity score (PS) matching and inverse probability treatment weighting (IPTW) were used and compared with a standard multivariable logistic regression model. Analyses were performed in complete cases (N = 4586) and in whole dataset with missing measurements imputed (N = 8199).

Method		Marginal OR (co-amoxiclav vs amoxicillin)	95 %CI	p-value
**Complete cases (N = 4586)**				
**PS Matching**	Univariable	0.92	0.67–1.25	0.59
	Adjusted for variables with SMD > 0.1	0.95	0.73–1.23	0.67
**IPTW**	Univariable	1.05	0.81–1.37	0.72
	Adjusted for variables with SMD > 0.1	1.02	0.78–1.33	0.87
**Multivariable logistic regression**		1.08	0.84–1.41	0.54
**Multiple imputation (N = 8199)**				
**PS Matching**	Univariable	1.10	0.84–1.45	0.48
	Adjusted for variables with SMD > 0.1	1.11	0.85–1.46	0.43
**IPTW**	Univariable	1.09	0.84–1.43	0.52
	Adjusted for variables with SMD > 0.1	1.11	0.86–1.43	0.42

Using a second causal approach with IPTWs, extreme weights were truncated at 99.9th percentiles to achieve good covariate balance (Table S5; Fig. S3 shows the distribution of weights after truncation). This approach also showed no evidence that 30-day mortality differed in patients initially receiving co-amoxiclav vs. amoxicillin (marginal OR 1.05 [0.81–1.37, p = 0.72]; with additional adjustment for imbalanced covariates, 1.02 [0.78–1.33, p = 0.87]) (Table 3). Results were consistent restricting to admissions with radiologically-confirmed pneumonia (Table S4B). Results were also consistent restricting to admissions with only oral or intravenous administration of antibiotics (Table S4C).

### Sensitivity analysis using imputation for missing data

After imputation of missing values in covariates with 1–21 % missingness (Table S6), the distribution of imputed values was broadly similar to that of original values (Fig. S4). Following PS matching and IPTW, mean covariate SMD across the 25 imputed datasets indicated successful balance (Tables S7, S8). There was no evidence of differences between co-amoxiclav vs. amoxicillin in pooled treatment effects across the imputations, although point estimates were slightly higher than complete case analyses (marginal OR range: 1.09–1.11) (Table 3), suggesting patients with missing values may have slightly different characteristics to those with complete measurements.

### Associations between covariates and 30-day mortality from logistic regression

Using cases with complete data (4586 admissions, 47.4 %), in a standard multivariable logistic regression, there was also no evidence of an association between 30-day all-cause mortality and receipt of co-amoxiclav only versus amoxicillin only (adjusted OR, aOR=1.08 [95 %CI 0.84–1.41], p = 0.54) (Table 3). Similarly, there was no evidence of differences in 30-day all-cause mortality associated with receipt of additional gentamicin (0.98 [0.71–1.34], p = 0.89), additional macrolides (1.13 [0.89–1.44], p = 0.33), or additional doxycycline (1.00 [0.72–1.38], p = 0.99), compared to those on amoxicillin/co-amoxiclav alone (Table S9).

Multiple other baseline factors were associated with all-cause 30-day mortality (Table S9). Older age (aOR=1.46 per 10 years older [95 %CI 1.27–1.70;p < 0.001]), palliative care (12.36 [9.20–16.71;p < 0.001]), higher oxygen flow rate (1.04 per 1 litre/minute higher, [1.01–1.08;p = 0.02]), respiratory rate (1.05 per 1 higher, [1.02–1.09;p = 0.02]), and lactate (1.23 per 1 mmol/L higher, [1.10–1.38;p = 0.001]) were associated with greater 30-day mortality, whereas higher albumin (0.91 per 1 g/L higher, [0.89–0.94;p < 0.001]) and being diagnosed with UTI in previous year (0.67 [0.47–0.96;p = 0.03]) were associated with lower 30-day mortality, the latter possibly reflecting miscoding of the current episode as CAP instead of UTI (which typically has better outcomes). Several factors also had non-linear associations with mortality (Figs. S5, S6). Overall, abnormal vital signs and blood measurements were associated with a higher mortality.

### Stratified analyses by pneumonia severity

Using CURB-65 scores, 2006 (22.2 %) patients had severe pneumonia (score 3–5), 3173 (35.0 %) moderate pneumonia (score 2), and 3881 (42.8 %) mild pneumonia (score 0–1) (Table 1). 625 patients had unknown severity due to missing CURB-65 score components. Unadjusted 30-day mortality in patients treated with co-amoxiclav vs. amoxicillin was 33.9 % vs 17.2 % in severe patients, 21.5 % vs 14.5 % in moderate patients, and 7.9 % vs 2.8 % in mild patients, respectively, reflecting clinicians identifying more serious infections and prescribing co-amoxiclav more frequently even within each severity subgroup.

After PS matching, effect estimates among severe, moderate, and mild patients were similar to the overall analyses, with no evidence of differences between receipt of initial co-amoxiclav vs. amoxicillin in adjusted models (severe: marginal OR=1.04 [0.77–1.42], p = 0.79; moderate: 0.93 [0.67–1.31], p = 0.69; mild: 0.96 [0.60–1.52], p = 0.85). Results remained consistent using IPTWs (severe: marginal OR=1.08 [0.78–1.50], p = 0.64; moderate: 1.02 [0.71–1.46], p = 0.92; mild: 1.04 [0.72–1.49], p = 0.47). Similarly, in logistic regression models with covariate adjustment, there was no evidence of an association between receipt of initial co-amoxiclav vs. amoxicillin in all three severity groups (Table 4). Results remained broadly similar in sensitivity analyses with missing data imputed, with point estimates being slightly higher (Table 4).

**Table 4:** Average treatment effects (marginal odds ratios (ORs) and 95 % confidence intervals (CIs)) of baseline co-amoxiclav vs amoxicillin on 30-day all-cause mortality in subgroup analyses stratified by baseline pneumonia severity. Severity was determined by CURB-65 score: severe pneumonia (score 3–5), moderate pneumonia (score 2), and mild pneumonia (score 0–1). Propensity score (PS) matching and inverse probability treatment weighting (IPTW) were used and compared with a standard multivariable logistic regression model. Analyses were performed in complete cases (N = 4586) and in whole dataset with missing measurements imputed (N = 7623, 576 were unknown severity due to missingness in CURB-65 score).

		Severe			Moderate				Mild	
Method		Marginal OR	95 %CI	p-value	Marginal OR	95 %CI	p-value	Marginal OR	95 %CI	p-value
**Complete cases (N = 4586)**		N = 1096			N = 1698				N = 1792	
**Matching**	Univariable	0.87	0.44–1.72	0.69	1.13	0.64–2.00	0.67	0.92	0.51–1.66	0.78
	Adjusted for variables with SMD > 0.1	1.04	0.77–1.42	0.79	0.93	0.67–1.31	0.69	0.96	0.60–1.52	0.85
**IPTW**	Univariable	1.05	0.41–1.68	0.92	1.07	0.76–1.51	0.71	1.04	0.72–1.49	0.85
	Adjusted for variables with SMD > 0.1	1.08	0.78–1.50	0.64	1.02	0.71–1.46	0.92	1.04	0.72–1.49	0.83
**Multivariable logistic regression**		0.90	0.55–1.49	0.69	1.12	0.77–1.63	0.54	1.24	0.70-2.24	0.47
**Multiple imputation (N = 7623)**		N = 1681			N = 2643				N = 3299	
**Matching**	Univariable	0.96	0.62–1.47	0.84	1.23	0.64–2.38	0.53	1.20	0.68–2.10	0.53
	Adjusted for variables with SMD > 0.1	1.08	0.76–1.54	0.66	1.14	0.81–1.61	0.45	1.22	0.72–2.07	0.47
**IPTW**	Univariable	1.00	0.57–1.78	0.98	1.18	0.90–1.56	0.22	1.21	0.75–1.96	0.44
	Adjusted for variables with SMD > 0.1	1.08	0.78–1.50	0.64	1.16	0.87–1.54	0.31	1.21	0.75–1.96	0.43

## Discussion

In this EHR study using causal inference methods to estimate the effects of initial antibiotic choices from observational data, we found no evidence of a difference in 30-day all-cause mortality between patients hospitalised with CAP initially treated with co-amoxiclav vs. amoxicillin. We tested several different approaches, and consistently found no evidence of a difference in mortality. We also found no evidence of an association between mortality and receiving additional gentamicin, macrolide, or doxycycline at baseline.

Point estimates comparing the impact of initial co-amoxiclav to amoxicillin on 30-day mortality ranged between 0.92 to 1.11; the estimated confidence intervals for the main PS matched analysis (95 %CI 0.73–1.23) indicate that we can exclude amoxicillin being associated with increased mortality by more than 27 % with reasonable confidence, and similarly that co-amoxiclav is not associated with increased mortality by more than 23 %, albeit with the most likely range of values closer to the point estimate (0.95).

Our findings are consistent with several earlier studies. A recent multi-centre randomised trial in the Netherlands showed narrow-spectrum treatment for moderately-severe CAP outside of the ICU was non-inferior to broad-spectrum treatment in terms of 90-day mortality.^28^ Although some previous studies using similar PS approaches reported increased mortality associated with broad-spectrum antibiotics for CAP and healthcare-associated pneumonia (HAP),^29^, ^30^, ^31^ these studies acknowledge this may arise from unmeasured confounders unavailable in their administrative databases, a limitation which we address by potentially adjusting more completely for confounding with our more detailed data.

Our study suggests wider use of narrow-spectrum agents may potentially be safe for the treatment of CAP requiring hospital admission. Across several thousand patients, within the assumptions made, there was no evidence that using amoxicillin rather than co-amoxiclav was harmful; while other data suggest this could reduce AMR^32^ and antibiotic-related adverse events.^33^ Empirical treatment based on clinical syndromes is by definition population-based, even if some stratification by disease severity is recommended, as in CAP guidelines (although, we did not find any evidence that our findings depended on pneumonia severity scores). However, clinicians and patients may be rightly concerned that they do not want to see individual patients deprived of potentially lifesaving treatment. This is common to all empirical antibiotic use, which balances such risks against AMR, rather than simply offering everyone the broadest possible, most likely to be active, treatment. Although our findings suggest that amoxicillin can probably be used to successfully treat more CAP than at present, better diagnostics to identify the causative organisms in CAP more often would also be useful, alongside other demographic and healthcare data, as a basis for generating personalised treatment recommendations, to ensure antibiotic resistant or atypical infections are appropriately treated.

Our study has several limitations. The causal estimates produced depend on several assumptions, including that all determinants of treatment choice are included in the models. However despite using comprehensive EHR data, unmeasured confounding likely still exists: there may be unwritten/unrecorded factors that lead clinicians to use broad-spectrum antibiotics in more unwell patients, even when all measured factors are the same (potentially undermining model assumptions). Hence, our study provides clear support for a future randomised trial that could be used to address this concern more completely; however, investment in such a trial would need to be balanced against how likely unmeasured confounding sufficient to change study findings was, and how generalisable findings might be over time and to different locations.

Other limitations include using diagnostic codes to identify CAP, which may be imperfect, although findings were similar in the subset of patients with radiological evidence of pneumonia. Additionally, we did not account for the causative organism, since, in common with most CAP, most patients (94 %) lacked positive microbiology. This also precluded subgroup analyses by causative pathogens. In the subset of patients with positive blood cultures (3.1 %), *S. aureus, K. pneumoniae* and ampicillin-resistant *H. influenzae* accounted for 76/298 (26 %) of infections, which would not be expected to respond to amoxicillin. However, as most patients did not have positive microbiology it is possible that beta-lactamase producing bacteria made up a smaller proportion of cases overall, which would be in keeping with our main findings suggesting amoxicillin was active in most cases. In settings with different bacterial species or resistance prevalence our findings may not generalise. As above, better diagnostics to reliably diagnose more of the infections that would not respond to amoxicillin, might help support wider use of narrow spectrum agents.

Testing in our setting for atypical causes of pneumonia was relatively sparse, 3.9 % were tested with a PCR capable of detecting *Mycoplasma* and 11.5 % had a *Legionella* urinary antigen test performed, with only 7 and 2 *Mycoplasma* and *Legionella* infections detected overall. The limited number of atypical infections likely explains the lack of association between additional macrolides or doxycycline and mortality, rather than these antibiotics being of no value in atypical pneumonia. However, there was no evidence that their relatively common use at a population level impacted outcomes overall.

Our primary analyses were restricted to the subset of patients with non-missing covariates, as this provides the best control for confounding under missing at random assumptions. However, sicker patients have a higher likelihood of having some measurements recorded, e.g. lactate, potentially limiting generalisability of findings to less unwell patients with more missing data. We therefore performed sensitivity analyses imputing missing values with broadly similar results. We did not adjust for smoking status; this was not available in our dataset. We did not adjust for individual clinicians, however their different prescribing preferences make the study possible. We only investigated baseline antibiotics rather than using a framework for accounting for changes in antibiotics over time. Only a very small proportion of patients were admitted to Intensive Care within the baseline period (1.7 %), and so further data is needed to make recommendations for this specific patient group. Also, we did not have data on antibiotic usage in the community prior to hospital admission.

In conclusion, among adults hospitalised with CAP, we found no evidence that co-amoxiclav provides an advantage over amoxicillin in 30-day mortality at a population-level. Given the potential for more AMR and adverse events with co-amoxiclav, the wider use of amoxicillin in empiric treatment of moderate/severe CAP should be considered and ideally be subject to a well-powered non-inferiority randomised control trial. Additionally, better diagnostics and data-driven tools are also needed to identify patients at risk of antibiotic resistant or atypical infections who may benefit from broader spectrum initial antibiotic treatment.

## Funding

This study was funded by the National Institute for Health Research (NIHR) Health Protection Research Unit in Healthcare Associated Infections and Antimicrobial Resistance at Oxford University in partnership with the UK Health Security Agency (UKHSA) and the NIHR Biomedical Research Centre, Oxford. ASW is also supported by core support from the Medical Research Council UK to the MRC Clinical Trials Unit [MC_UU_12023/22] and is an NIHR Senior Investigator. DWE is supported by a Robertson Fellowship. The views expressed in this publication are those of the authors and not necessarily those of the NHS, the National Institute for Health Research, the Department of Health or the UKHSA.

## CRediT authorship contribution statement

The study was designed and planned by DWE, ASW, NMR, and NJ. The specific analysis was designed by JW, DWE, and ASW. JW, AU, CN, HW, YI, QG, HY contributed to the statistical analysis of the data. JW, DWE, and ASW drafted the manuscript and all authors contributed to interpretation of the data and results and revised the manuscript. All authors approved the final version of the manuscript.

## Declaration of Competing Interest

The authors declare that they have no known competing financial interests or personal relationships that could have appeared to influence the work reported in this paper.

## Data Availability

The datasets analysed during the current study are not publicly available as they contain personal data but are available from the Infections in Oxfordshire Research Database (https://oxfordbrc.nihr.ac.uk/research-themes-overview/antimicrobial-resistance-and-modernising-microbiology/infections-in-oxfordshire-research-database-iord/), subject to an application and research proposal meeting the ethical and governance requirements of the Database. For further details on how to apply for access to the data and for a research proposal template please email iord@ndm.ox.ac.uk.
