# Supplementary material for: No evidence of difference in mortality with amoxicillin versus co-amoxiclav for hospital treatment of community-acquired pneumonia
Source: J Infect. 2024 Jun;88(6):None. doi: 10.1016/j.jinf.2024.106161 (PMC11893475; doi:10.1016/j.jinf.2024.106161)
Supplement: Supplementary file 1 — Supplementary material [file mmc1.docx]

## No evidence of difference in mortality with amoxicillin versus co-amoxiclav for hospital treatment of community-acquired pneumonia: Supplementary Methods

### Microbiology and radiology data

Microbiology test results were linked to CAP admission to identify the proportion of admissions with a pneumonia pathogen isolated from blood culture, including *Streptococcus pneumoniae*, *Pseudomonas aeruginosa, Staphylococcus aureus,* *Mycoplasma pneumoniae*, *Legionella pneumophila*, *Klebsiella pneumoniae, Haemophilus influenzae,* *Moraxella catarrhalis*, and *Chlamydia pneumoniae.* Positive results for Legionella urinary antigen test, influenza/RSV PCR, and multiplex respiratory PCR were also reported.

Radiology chest X-ray (CXR) reports and chest/pulmonary/thorax/aorta CT scans were linked to each admission. Presence of pneumonia was identified using fuzzy string matching if the CXR/CT report contained the following keywords: 'pneumonia', 'consolidation', 'infiltrate', 'airspace', 'bronchopneumonia', 'infection', 'infective', 'air bronchogram', 'density', 'pneumonic', 'abscess', 'aspiration', 'cavity', and the keyword was not negated (negation detection was conducted using negspaCy package in Python, which is based on the NegEx algorithm for identifying negated findings in medical records^1^).

### Exposures

Charlson comorbidity score and hospital frailty risk score were calculated using all inpatient and outpatient diagnosis codes from the year prior to admission and all secondary diagnosis codes from the current admission. Additional specific comorbidities that were potential predictors of mild/severe infection or lower/greater likelihood of mortality were additionally adjusted for, using the same set of diagnostic codes as above: i) urinary tract infection (UTI): N300, N301, N302,N303, N305, N309, N340, N342, N390, N410, N411, N412, N413, N131, N132, N133, N134, N136, N419, O230, O233, O239, P393, R300, R309; ii) immunosuppression: B20–24 (AIDS/HIV), 0987, C77-96 (metastatic cancer, haematological malignancies), D80-84 (primary immunodeficiencies), K721, K729, K766, K767 (end-stage liver disease); iii) palliative care: Z515; iv) autoimmune diseases^2^: D510, D591, D693, E050, E063, E10, E271, G35, G610, H20, K50, K51, K73, K743, K900, L10, L12, L40, L63, L809, M05, M06, M08, M313, M33, M315, M316, M353, G700, M34, M321, M329, M350, M459.

CURB-65 scores were calculated by summing one point for each of the following: confusion, urea >7mmol/L, respiratory rate ≥30 breaths/min, blood pressure <90mmHg (systolic) or ≤60mmHg (diastolic), age ≥65 years. Calculations used available baseline vital signs, considering patients with an AVPU status other than alert or a Glasgow Coma Scale (GCS) <15 as confused. Patients with missing measurements of urea, respiratory rate, systolic blood pressure, or diastolic blood pressure were classified as ‘unknown’.

We additionally adjusted for SMART-COP score^3,4^ and PSI/PORT score^5,6^ for pneumonia severity. SMART-COP score was calculated by summing the following: multi-lobar involvement on chest X-ray (1), albumin <35 d/L (1), respiratory rate ≥25 breaths/min if age ≤ 50; ≥30 breaths/min if age >50 (1), heart rate ≥125 beats/min (1), confusion (1), PaO₂ <70 mmHg or oxygen saturation ≤93% or ratio of arterial partial pressure of oxygen to fraction of inspired oxygen (PaO₂/FiO₂) <333 if age ≤50; PaO₂ <60 mmHg or oxygen saturation ≤90% or PaO₂/FiO₂ <250 if age >50 (2), pH<7.35 (2), systolic blood pressure <90 mmHg (2). PSI/PORT score was calculated by summing the following: age (points=age), sex (-10 if female, 0 if male), nursing home resident (10), neoplastic disease (30), liver disease history (20), congestive heart failure history (10), cerebrovascular disease history (10), altered mental status (defined as confusion as above, 20), respiratory rate ≥30 breaths/min (20), systolic blood pressure <90mmHg (20), temperature <35°C or >39.9°C (15), heart rate ≥125 beats/min (10), pH<7.35 (30), urea ≥11mmol/L (20), sodium <130 mmol/L (20), glucose ≥14 mmol/L (10), hematocrit <30% (10), partial pressure of oxygen (PaO₂) <8 kPa (10), pleural effusion on X-ray (10). Components with missing values were assumed to be 0 score in complete case analyses and determined by imputed values in sensitivity analyses.

### Statistical methods

Across all models, continuous variables were truncated at the 2.5% and 97.5% percentiles to avoid undue influence from outliers. Non-linear effects of continuous variables were modelled using natural cubic splines where non-linear terms improved the model fit by Bayesian Information Criterion (BIC)>6 ^7^ in univariable models for 30-day all-cause mortality. The optimal number of knots (2-5) was chosen based on minimising the BIC. The same transformation was then used in all outcome and treatment models. Pairwise interactions between main effects were retained in final models if they improved the model fit by a BIC>6^7^.

All analyses were performed in R 4.1 using the following packages: tidyverse (version 1.3.2), survey (version 4.2-1), mice (version 3.16.0), MatchIt (version 4.5.4), WeightIt (version 0.14.2), MatchThem (version 1.1.0), comorbidity (version 1.0.5), marginaleffects (version 0.13.0), cobalt (version 4.5.1), tmle (version 2.0.0).

## References

1 Chapman WW, Bridewell W, Hanbury P, Cooper GF, Buchanan BG. A simple algorithm for identifying negated findings and diseases in discharge summaries. *J Biomed Inform* 2001; **34**: 301–10.

2 Eaton WW, Pedersen MG, Atladóttir HÓ, Gregory PE, Rose NR, Mortensen PB. The prevalence of 30 ICD-10 autoimmune diseases in Denmark. *Immunol Res* 2010; **47**: 228.

3 SMART-COP Score for Pneumonia Severity. https://www.mdcalc.com/calc/3914/smart-cop-score-pneumonia-severity (accessed Jan 12, 2024).

4 Charles P, Wolfe R, Whitby M, *et al.* SMART-COP: A tool for predicting the need for intensive respiratory or vasopressor support in community-acquired pneumonia. *Clinical Infectious Diseases* 2008; **47**: 375–84.

5 PSI/PORT Score: Pneumonia Severity Index for CAP. https://www.mdcalc.com/calc/33/psi-port-score-pneumonia-severity-index-cap (accessed Jan 12, 2024).

6 Fine MJ, Auble TE, Yealy DM, *et al.* A Prediction Rule to Identify Low-Risk Patients with Community-Acquired Pneumonia. *https://doi.org/101056/NEJM199701233360402* 1997; **51**: 834.

7 Raftery AE. Bayesian Model Selection in Social Research. *Sociol Methodol* 1995; **25**: 111.

## Supplementary Results


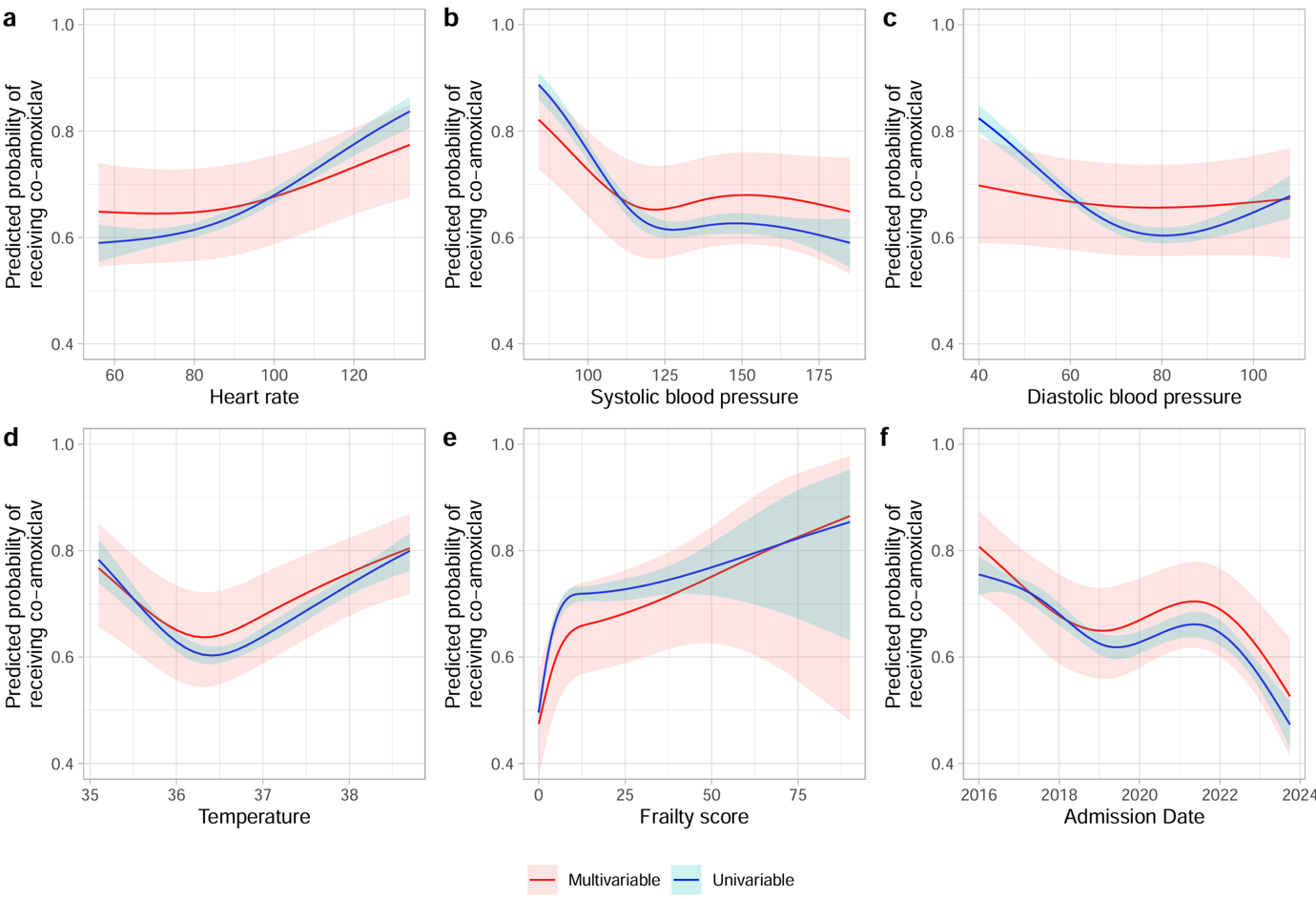


**Figure S1. Unadjusted and adjusted associations between antibiotic treatment (co-amoxiclav vs. amoxicillin) and vital signs (a-d), frailty scores (e), admission date (f) with non-linear effects.** The 95% CIs are calculated as estimates ± 1.96 × standard error of the estimates. Estimates for linear effects and categorical variables are shown in **Table 2**.


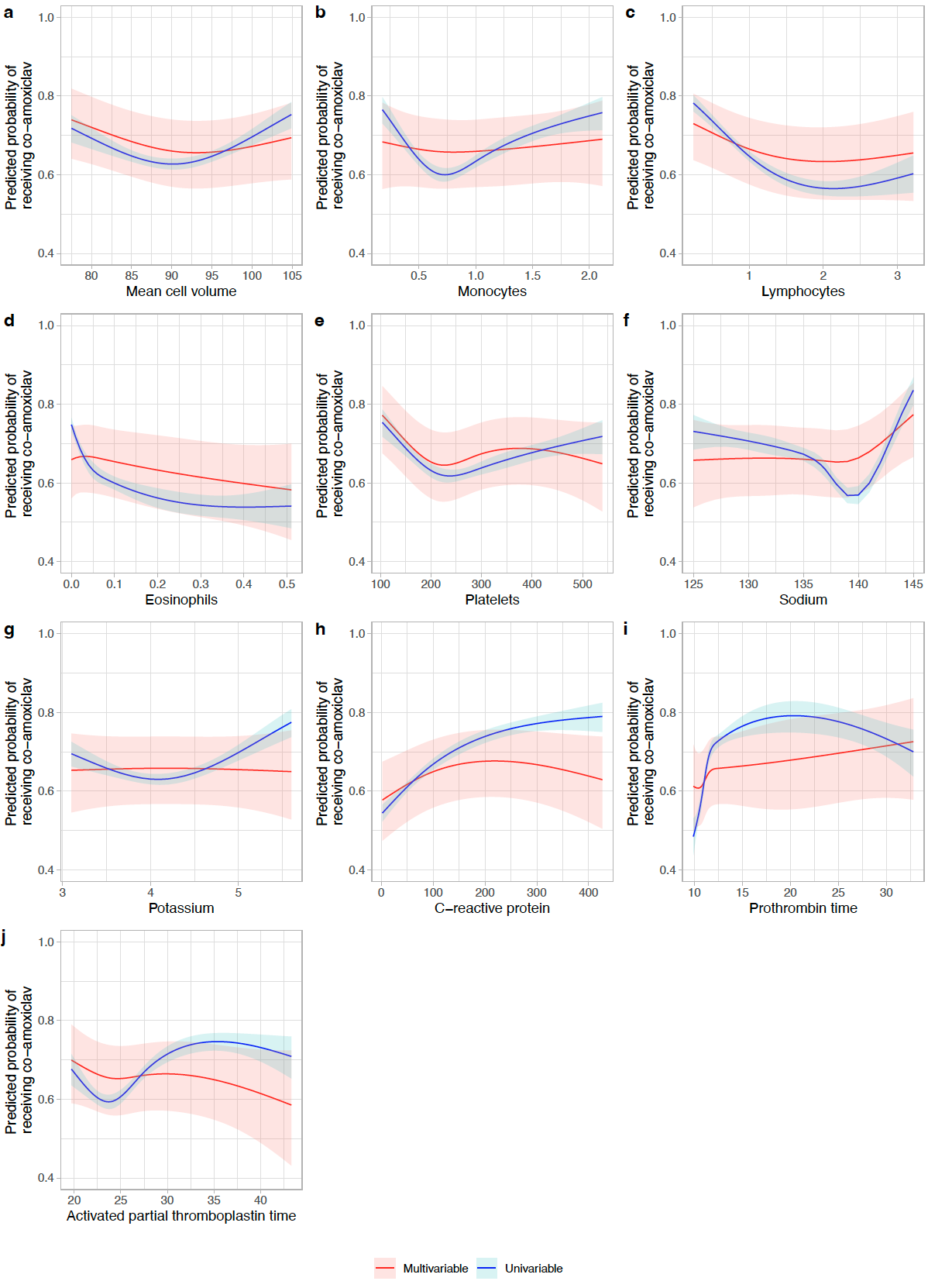


**Figure S2. Unadjusted and adjusted associations between antibiotic treatment (co-amoxiclav vs. amoxicillin) and laboratory measurements with non-linear effects.** The 95% CIs are calculated as estimates ± 1.96 × standard error of the estimates. Estimates for linear effects and categorical variables are shown in **Table 2**.


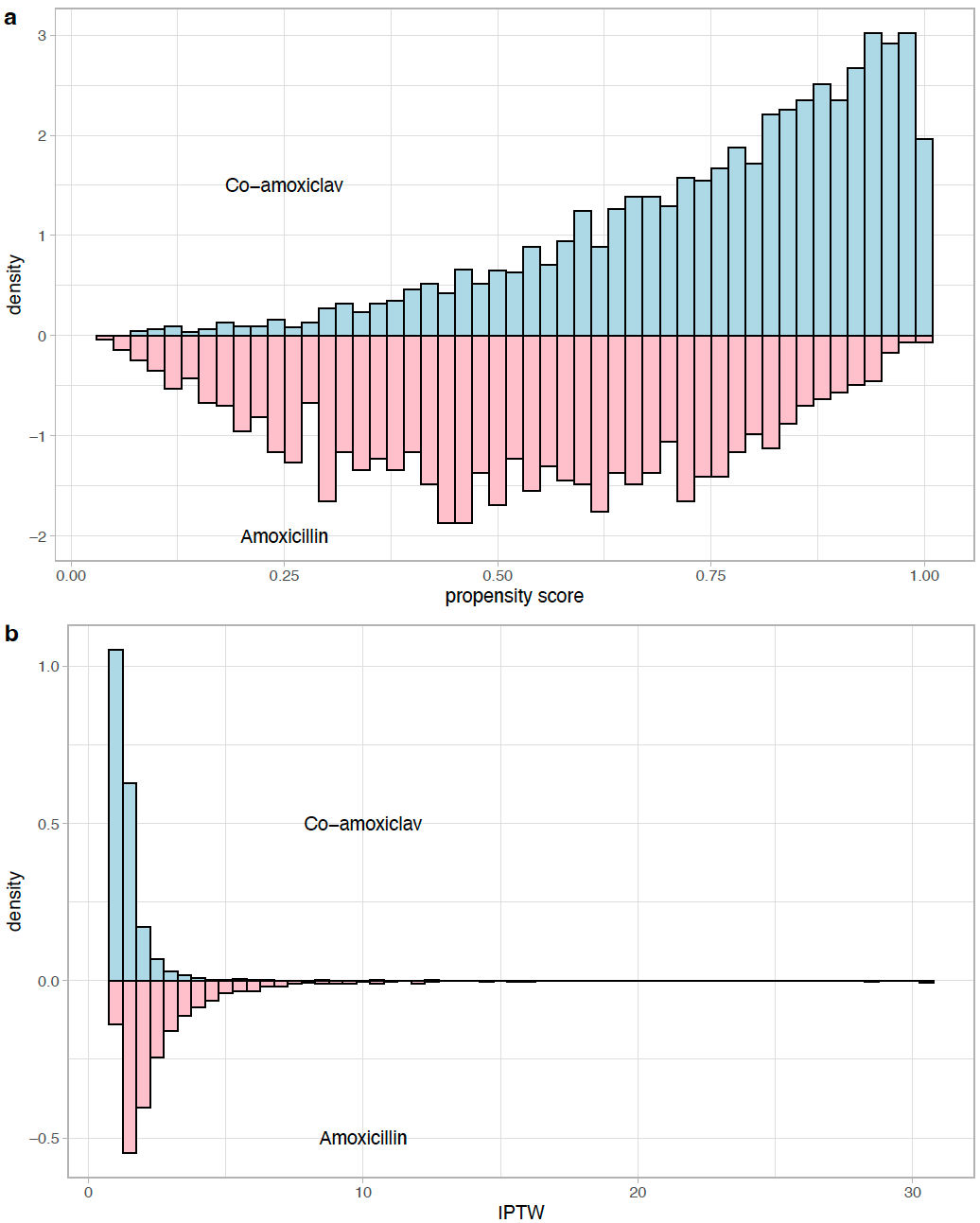


**Figure S3. Distribution of propensity scores (panel a) and inverse probability weights (panel b) by baseline treatment (co-amoxiclav or amoxicillin).**


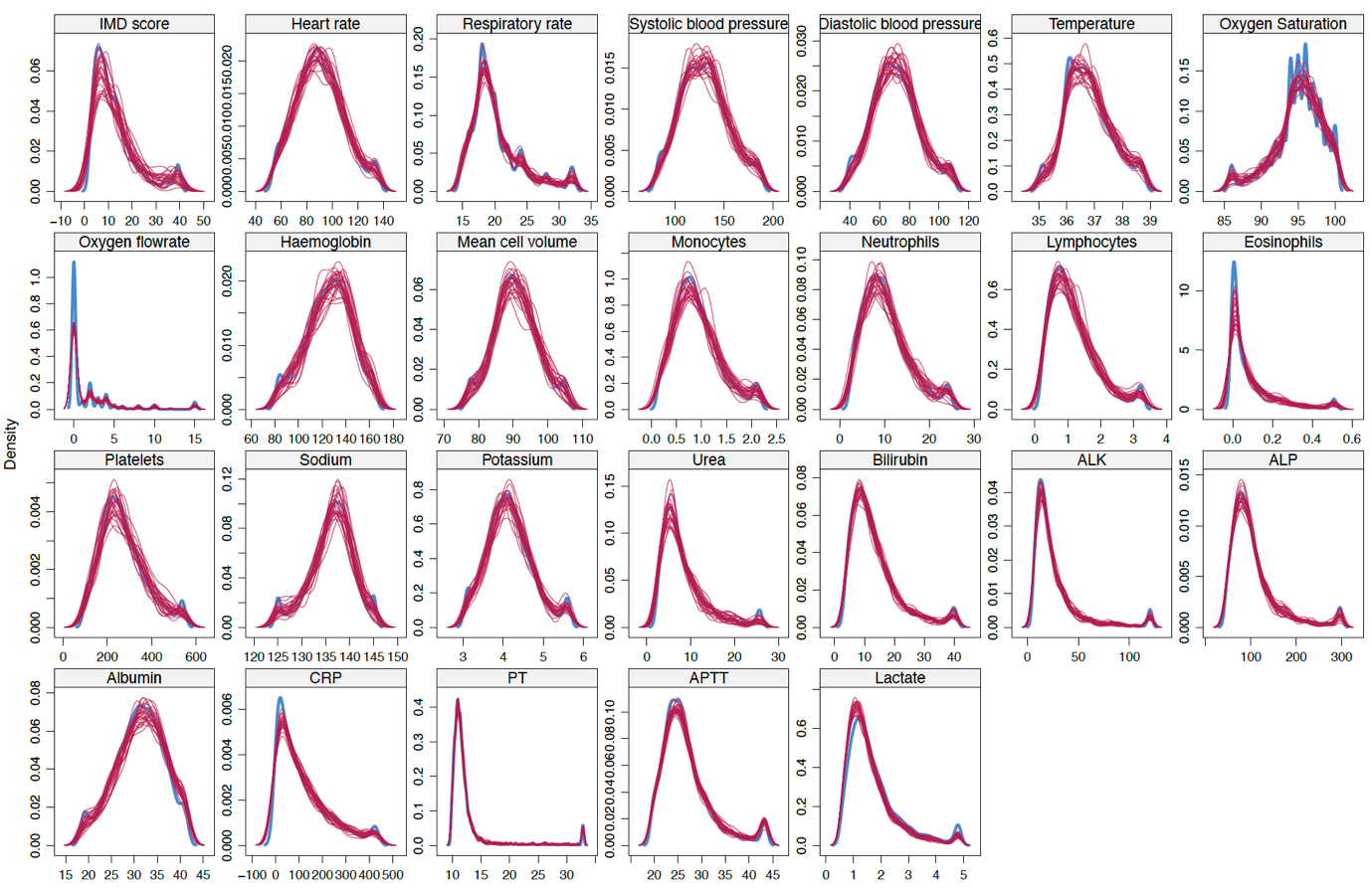


**Figure S4. Distribution of original measurements (blue line) and imputed measurements (red lines) for variables with missing values using multiple imputation with chain equation (MICE).** 25 imputed datasets were generated. IMD: index of multiple deprivation; SBP: systolic blood pressure; DBP: diastolic blood pressure; MCV: mean cell volume; ALT: alanine transferase; ALP: alkaline phosphatase; CRP: C-reactive protein; PT: prothrombin time; APTT: activated partial thromboplastin time.


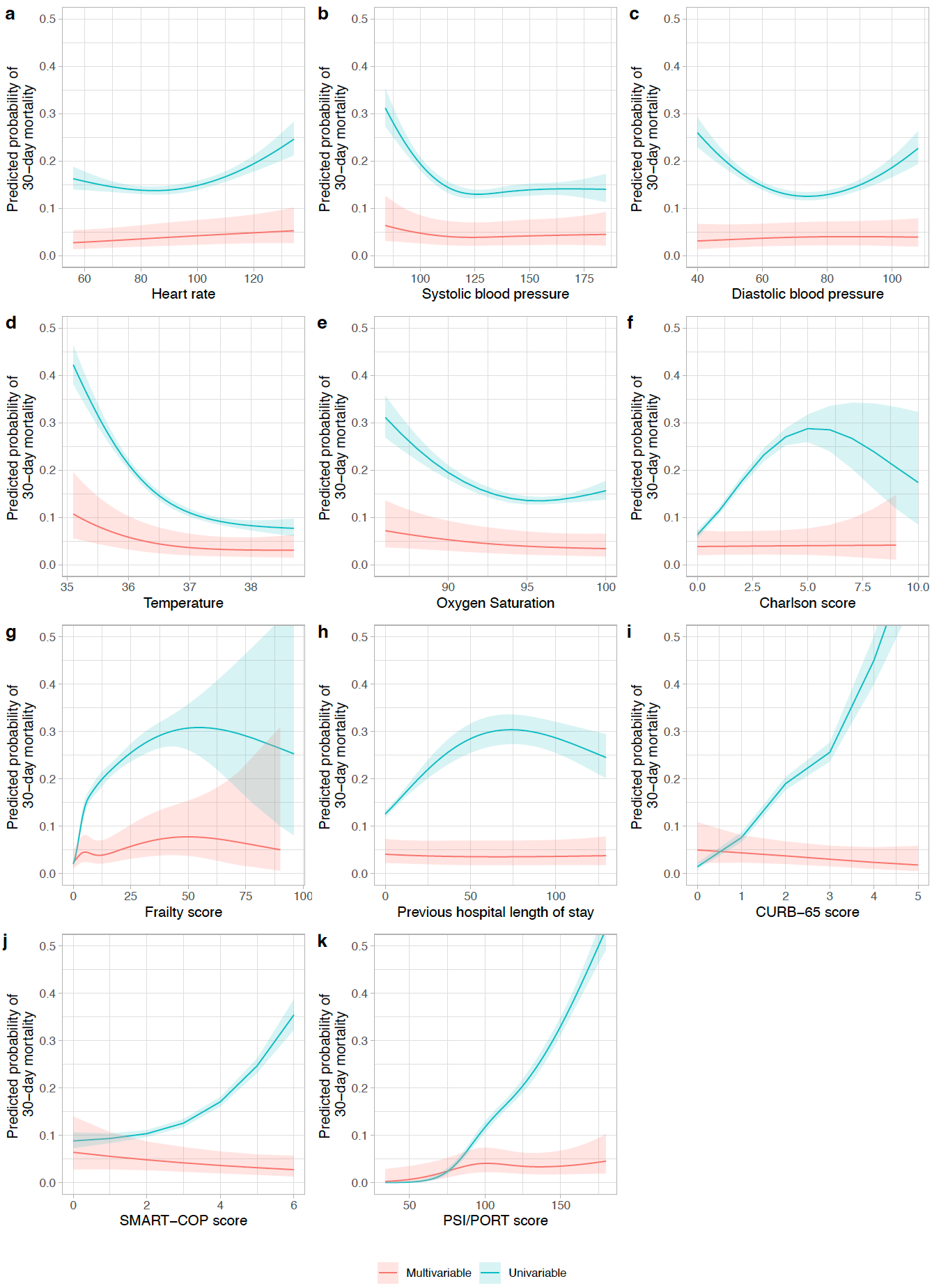


**Figure S5. Unadjusted and adjusted associations between 30-day all-cause mortality and vital signs (a-e), comorbidity scores (f-g), hospital length of stay in previous year (h), and pneumonia risk scores (i-k) with non-linear effects.** The 95% CIs are calculated as estimates ± 1.96 × standard error of the estimates. Estimates for linear effects and categorical variables are shown in **Table S9**.


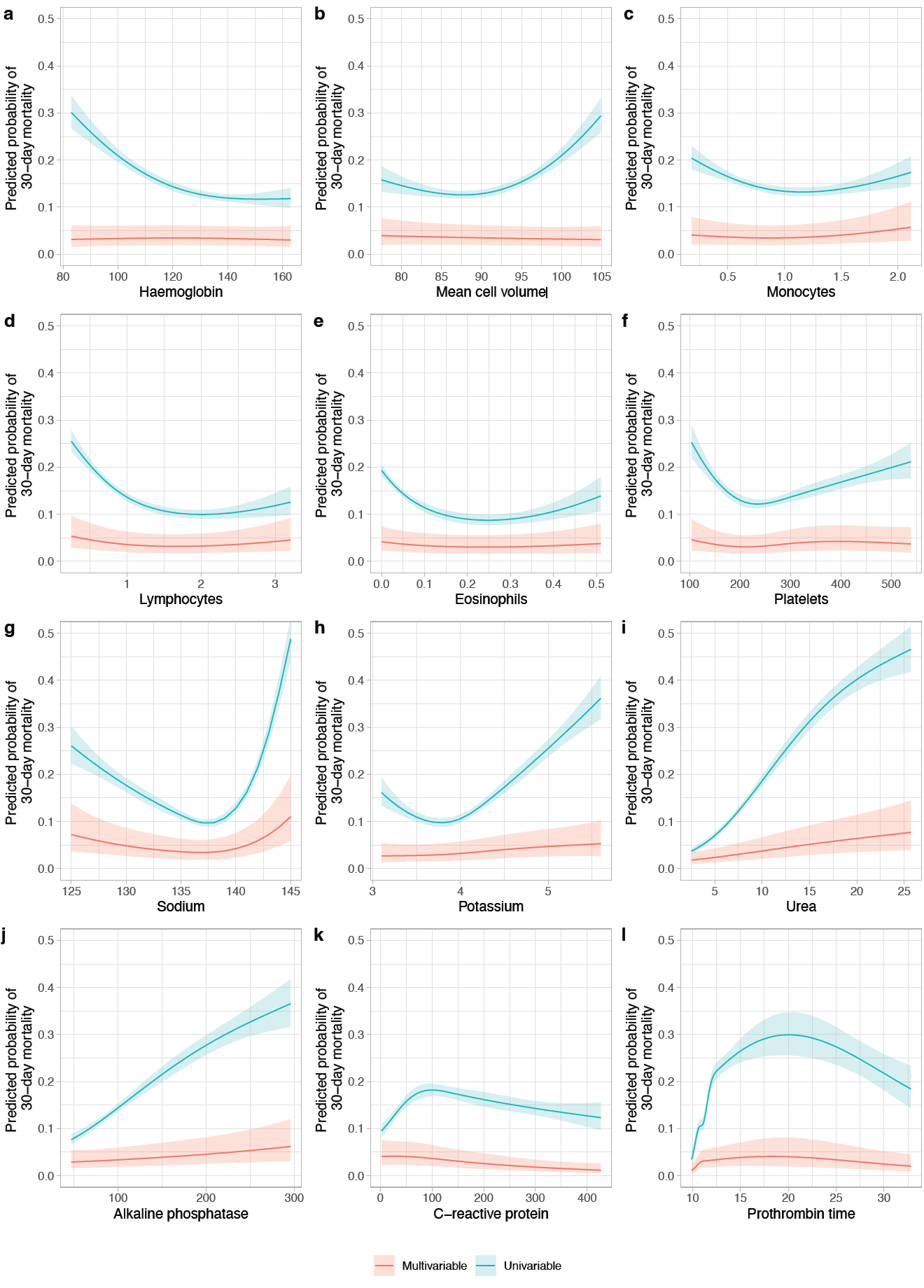


**Figure S6. Unadjusted and adjusted associations between 30-day all-cause mortality and laboratory measurements with non-linear effects.** The 95% CIs are calculated as estimates ± 1.96 × standard error of the estimates. Estimates for linear effects and categorical variables are shown in **Table S9**.

| Antibiotics received at baseline | Number | Proportion (%) |
| --- | --- | --- |
| Co-amoxiclav | 9376 | 65.73 |
| Amoxicillin | 5565 | 39.01 |
| Clarithromycin | 4477 | 31.39 |
| Doxycycline | 3146 | 22.06 |
| Ceftriaxone | 2448 | 17.16 |
| Gentamicin | 1416 | 9.93 |
| Piperacillin + tazobactam | 577 | 4.05 |
| Metronidazole | 312 | 2.19 |
| Moxifloxacin | 265 | 1.86 |
| Ciprofloxacin | 117 | 0.82 |
| Co-trimoxazole | 115 | 0.81 |
| Vancomycin | 87 | 0.61 |
| Nitrofurantoin | 75 | 0.53 |
| Meropenem | 70 | 0.49 |
| Flucloxacillin | 53 | 0.37 |
| Ceftazidime | 40 | 0.28 |
| Azithromycin | 38 | 0.27 |
| Trimethoprim | 36 | 0.25 |
| Piperacillin-tazobactam | 34 | 0.24 |
| Clindamycin | 24 | 0.17 |
| Phenoxymethylpenicillin | 20 | 0.14 |
| Cefalexin | 14 | 0.10 |
| Ertapenem | 12 | 0.08 |
| Benzylpenicillin | 5 | 0.04 |
| Lymecycline | 5 | 0.04 |
| Teicoplanin | 5 | 0.04 |
| Erythromycin | 4 | 0.03 |
| Oxytetracycline | 4 | 0.03 |
| Daptomycin | 3 | 0.02 |
| Colistin | 2 | 0.01 |
| Levofloxacin | 2 | 0.01 |
| Rifampicin | 2 | 0.01 |
| Amikacin | 1 | 0.01 |
| Aztreonam | 1 | 0.01 |
| Cefazolin | 1 | 0.01 |
| Cefuroxime | 1 | 0.01 |
| Chloramphenicol | 1 | 0.01 |
| Fosfomycin | 1 | 0.01 |
| Fusidic acid | 1 | 0.01 |
| Linezolid | 1 | 0.01 |
| Phenoxymethylpenicillin potassium | 1 | 0.01 |
| Pivmecillinam | 1 | 0.01 |

**Table S1. Frequency of baseline antibiotics received among 14,264 CAP patients who had antibiotic prescription at baseline.** Patients who did not receive either amoxicillin or co-amoxiclav at baseline, and those who received antibiotics other than amoxicillin, co-amoxiclav, macrolides, doxycycline, and gentamicin at baseline were excluded from the analyses (**Figure 1**).

|  | Amoxicillin (N=2841) | Co-amoxiclav (N=5358) | Both (N=1486) | Total (N=9685) | p value |
| --- | --- | --- | --- | --- | --- |
| Heart rate |  |  |  |  | < 0.001 |
| Median (Q1, Q3) | 86.0 (75.0, 98.8) | 91.0 (78.0, 104.0) | 89.0 (78.0, 101.0) | 89.0 (77.0, 102.0) |  |
| Missing, N | 167 | 223 | 27 | 417 |  |
| Respiratory rate |  |  |  |  | < 0.001 |
| Median (Q1, Q3) | 19.0 (17.0, 20.0) | 19.0 (18.0, 23.0) | 19.0 (18.0, 22.0) | 19.0 (18.0, 22.0) |  |
| Missing, N | 167 | 223 | 27 | 417 |  |
| Systolic blood pressure |  |  |  |  | < 0.001 |
| Median (Q1, Q3) | 132.0 (116.0, 148.0) | 126.0 (109.0, 144.0) | 127.0 (111.0, 145.0) | 128.0 (111.0, 145.0) |  |
| Missing, N | 167 | 223 | 27 | 417 |  |
| Diastolic blood pressure |  |  |  |  | < 0.001 |
| Median (Q1, Q3) | 72.0 (62.0, 81.0) | 68.0 (58.0, 79.0) | 68.0 (58.0, 80.0) | 69.0 (59.0, 80.0) |  |
| Missing, N | 167 | 223 | 27 | 417 |  |
| Temperature |  |  |  |  | < 0.001 |
| Median (Q1, Q3) | 36.6 (36.1, 37.1) | 36.7 (36.1, 37.4) | 36.8 (36.2, 37.4) | 36.7 (36.2, 37.3) |  |
| Missing, N | 167 | 223 | 27 | 417 |  |
| Saturation |  |  |  |  | < 0.001 |
| Median (Q1, Q3) | 96.0 (94.0, 97.0) | 95.0 (93.0, 97.0) | 95.0 (94.0, 97.0) | 95.0 (94.0, 97.0) |  |
| Missing, N | 184 | 268 | 38 | 490 |  |
| Use of oxygen supplement |  |  |  |  | < 0.001 |
| No | 2002 (74.9%) | 2904 (56.6%) | 813 (55.7%) | 5719 (61.7%) |  |
| Yes | 672 (25.1%) | 2231 (43.4%) | 646 (44.3%) | 3549 (38.3%) |  |
| Missing, N | 167 | 223 | 27 | 417 |  |
| Oxygen flow rate |  |  |  |  | < 0.001 |
| Median (Q1, Q3) | 3 (2, 4) | 3 (2, 6) | 3 (2, 4) | 3 (2, 5) |  |
| Missing, N | 167 | 223 | 27 | 417 |  |
| AVPU |  |  |  |  | < 0.001 |
| Alert | 2761 (97.2%) | 4857 (90.6%) | 1409 (94.8%) | 9027 (93.2%) |  |
| Voice | 79 (2.8%) | 437 (8.2%) | 75 (5.0%) | 591 (6.1%) |  |
| Pain | 1 (0.0%) | 46 (0.9%) | 2 (0.1%) | 49 (0.5%) |  |
| Unresponsive | 0 (0.0%) | 18 (0.3%) | 0 (0.0%) | 18 (0.2%) |  |
| NEWS2 score |  |  |  |  | < 0.001 |
| Median (Q1, Q3) | 2.0 (2.0, 5.0) | 5.0 (2.0, 7.0) | 5.0 (2.0, 7.0) | 4.0 (2.0, 7.0) |  |
| Missing, N | 184 | 268 | 38 | 490 |  |
| Haemoglobin |  |  |  |  | < 0.001 |
| Median (Q1, Q3) | 129.0 (115.0, 140.2) | 125.0 (111.0, 138.0) | 127.0 (114.0, 139.0) | 127.0 (113.0, 139.0) |  |
| Missing, N | 73 | 126 | 24 | 223 |  |
| Mean cell volume |  |  |  |  | 0.22 |
| Median (Q1, Q3) | 90.4 (86.8, 94.2) | 90.5 (86.5, 95.1) | 90.5 (86.7, 94.5) | 90.5 (86.6, 94.7) |  |
| Missing, N | 73 | 128 | 24 | 225 |  |
| Monocytes |  |  |  |  | 0.002 |
| Median (Q1, Q3) | 0.8 (0.6, 1.1) | 0.8 (0.6, 1.2) | 0.9 (0.6, 1.2) | 0.8 (0.6, 1.1) |  |
| Missing, N | 83 | 147 | 29 | 259 |  |
| Neutrophils |  |  |  |  | < 0.001 |
| Median (Q1, Q3) | 8.3 (5.9, 11.2) | 10.1 (7.0, 13.9) | 10.1 (7.3, 13.6) | 9.5 (6.7, 13.1) |  |
| Missing, N | 83 | 147 | 29 | 259 |  |
| Lymphocytes |  |  |  |  | 0.58 |
| Median (Q1, Q3) | 1.1 (0.8, 1.7) | 0.9 (0.6, 1.4) | 0.9 (0.6, 1.4) | 1.0 (0.6, 1.5) |  |
| Missing, N | 83 | 147 | 29 | 259 |  |
| Eosinophils |  |  |  |  | < 0.001 |
| Median (Q1, Q3) | 0.1 (0.0, 0.2) | 0.0 (0.0, 0.1) | 0.0 (0.0, 0.1) | 0.0 (0.0, 0.1) |  |
| Missing, N | 84 | 147 | 30 | 261 |  |
| Platelets |  |  |  |  | < 0.001 |
| Median (Q1, Q3) | 253.0 (202.0, 319.0) | 254.0 (195.0, 328.0) | 239.5 (189.0, 304.2) | 252.0 (196.0, 322.0) |  |
| Missing, N | 74 | 132 | 26 | 232 |  |
| Sodium |  |  |  |  | 0.02 |
| Median (Q1, Q3) | 137.0 (134.0, 139.0) | 136.0 (133.0, 139.0) | 137.0 (133.0, 139.0) | 137.0 (134.0, 139.0) |  |
| Missing, N | 72 | 122 | 23 | 217 |  |
| Potassium |  |  |  |  | 0.003 |
| Median (Q1, Q3) | 4.1 (3.8, 4.4) | 4.1 (3.8, 4.5) | 4.1 (3.7, 4.4) | 4.1 (3.8, 4.5) |  |
| Missing, N | 140 | 230 | 60 | 430 |  |
| Urea |  |  |  |  | < 0.001 |
| Median (Q1, Q3) | 6.2 (4.5, 9.1) | 7.4 (5.2, 11.3) | 7.1 (5.1, 10.2) | 7.0 (4.9, 10.5) |  |
| Missing, N | 73 | 125 | 23 | 221 |  |
| Bilirubin |  |  |  |  | < 0.001 |
| Median (Q1, Q3) | 11.0 (8.0, 16.0) | 11.0 (8.0, 17.0) | 12.0 (8.0, 18.0) | 11.0 (8.0, 17.0) |  |
| Missing, N | 243 | 410 | 120 | 773 |  |
| Alanine transferase |  |  |  |  | < 0.001 |
| Median (Q1, Q3) | 18.0 (13.0, 28.0) | 19.0 (12.0, 31.0) | 18.0 (12.0, 28.0) | 18.0 (12.0, 30.0) |  |
| Missing, N | 245 | 410 | 120 | 775 |  |
| Alkaline phosphatase |  |  |  |  | < 0.001 |
| Median (Q1, Q3) | 87.0 (70.0, 111.0) | 94.0 (73.0, 124.0) | 89.0 (71.0, 118.0) | 91.0 (72.0, 120.0) |  |
| Missing, N | 234 | 391 | 113 | 738 |  |
| Albumin |  |  |  |  | < 0.001 |
| Median (Q1, Q3) | 33.0 (29.0, 36.0) | 30.0 (27.0, 34.0) | 32.0 (28.0, 35.0) | 31.0 (28.0, 35.0) |  |
| Missing, N | 226 | 370 | 107 | 703 |  |
| C-reactive protein |  |  |  |  | < 0.001 |
| Median (Q1, Q3) | 60.4 (20.3, 134.3) | 96.3 (37.5, 187.3) | 90.5 (36.1, 188.8) | 84.5 (30.6, 173.1) |  |
| Missing, N | 208 | 348 | 89 | 645 |  |
| Prothrombin time |  |  |  |  | < 0.001 |
| Median (Q1, Q3) | 11.1 (10.6, 11.9) | 11.5 (10.9, 12.5) | 11.4 (10.8, 12.3) | 11.4 (10.8, 12.3) |  |
| Missing, N | 414 | 821 | 199 | 1434 |  |
| Activated partial thromboplastin time | |  |  |  | < 0.001 |
| Median (Q1, Q3) | 25.1 (23.1, 27.7) | 25.8 (23.4, 29.1) | 25.7 (23.5, 28.7) | 25.5 (23.3, 28.6) |  |
| Missing, N | 427 | 862 | 205 | 1494 |  |
| Lactate |  |  |  |  | < 0.001 |
| Median (Q1, Q3) | 1.4 (1.0, 1.9) | 1.6 (1.1, 2.2) | 1.5 (1.1, 2.1) | 1.5 (1.1, 2.1) |  |
| Missing, N | 816 | 927 | 162 | 1905 |  |
| pH |  |  |  |  | < 0.001 |
| >=7.25 | 2827 (99.5%) | 5204 (97.1%) | 1476 (99.3%) | 9507 (98.2%) |  |
| <7.25 | 14 (0.5%) | 154 (2.9%) | 10 (0.7%) | 178 (1.8%) |  |
| Severity by SMART-COP score |  |  |  |  | **< 0.001** |
| Low (0-2) | 408 (22.9%) | 501 (13.1%) | 193 (16.7%) | 1102 (16.3%) |  |
| Moderate (3-4) | 1185 (66.6%) | 2380 (62.4%) | 767 (66.3%) | 4332 (64.2%) |  |
| High (5-6) | 178 (10.0%) | 788 (20.6%) | 182 (15.7%) | 1148 (17.0%) |  |
| Very high (≥7) | 7 (0.4%) | 147 (3.9%) | 15 (1.3%) | 169 (2.5%) |  |
| Missing, N | 1063 | 1542 | 329 | 2934 |  |
| Severity by PSI/PORT score |  |  |  |  | **< 0.001** |
| Low_II (≤70) | 289 (15.4%) | 408 (9.8%) | 153 (12.0%) | 850 (11.6%) |  |
| Low_III (71-90) | 348 (18.5%) | 644 (15.5%) | 233 (18.3%) | 1225 (16.7%) |  |
| Moderate_IV (91-130) | 877 (46.7%) | 1841 (44.2%) | 581 (45.6%) | 3299 (45.1%) |  |
| High_V (>130) | 363 (19.3%) | 1275 (30.6%) | 307 (24.1%) | 1945 (26.6%) |  |
| Missing, N | 964 | 1190 | 212 | 2366 |  |

**Table S2. Baseline vital signs and laboratory measurements by initial antibiotics received.**

|  | Raw difference | Standardized mean difference | Balance |
| --- | --- | --- | --- |
| distance | 1.191 | 0.000 | Balanced, <0.1 |
| Age | 0.045 | -0.077 | Balanced, <0.1 |
| Sex-Male | 0.115 | 0.038 | Balanced, <0.1 |
| Ethnicity-Non-white | 0.025 | 0.069 | Balanced, <0.1 |
| IMD | 0.054 | 0.040 | Balanced, <0.1 |
| Additional gentamicin | 0.469 | -0.096 | Balanced, <0.1 |
| Additional macrolide | 0.236 | -0.153 | Not Balanced, >0.1 |
| Additional doxycycline | -0.580 | -0.007 | Balanced, <0.1 |
| Consultant-Acute medicine | 0.095 | -0.035 | Balanced, <0.1 |
| Consultant-Emergency Medicine | -0.173 | 0.008 | Balanced, <0.1 |
| Consultant-Gerontology | -0.015 | -0.023 | Balanced, <0.1 |
| Consultant-Infectious disease | -0.062 | 0.027 | Balanced, <0.1 |
| Consultant-Other | 0.042 | 0.048 | Balanced, <0.1 |
| Charlson score1 | 0.170 | -0.018 | Balanced, <0.1 |
| Charlson score2 | -0.115 | 0.017 | Balanced, <0.1 |
| Frailty score1 | 0.215 | 0.039 | Balanced, <0.1 |
| Frailty score2 | 0.121 | -0.026 | Balanced, <0.1 |
| Frailty score3 | 0.072 | 0.018 | Balanced, <0.1 |
| Frailty score4 | -0.001 | -0.036 | Balanced, <0.1 |
| Previous length of stay1 | 0.180 | -0.028 | Balanced, <0.1 |
| Previous length of stay2 | 0.029 | -0.057 | Balanced, <0.1 |
| Admissions in previous year | -0.113 | -0.035 | Balanced, <0.1 |
| Palliative care | 0.201 | -0.054 | Balanced, <0.1 |
| UTI | 0.106 | -0.023 | Balanced, <0.1 |
| Immunosuppression | 0.146 | -0.054 | Balanced, <0.1 |
| Autoimmune diseases | 0.016 | -0.053 | Balanced, <0.1 |
| Admission Date | -0.186 | 0.063 | Balanced, <0.1 |
| Admission hour:0-8 | -0.053 | -0.061 | Balanced, <0.1 |
| Admission hour:8-11 | 0.133 | 0.028 | Balanced, <0.1 |
| Admission hour:11-15 | -0.022 | 0.002 | Balanced, <0.1 |
| Admission hour:15-24 | -0.047 | 0.027 | Balanced, <0.1 |
| Admission: Wednesday | -0.027 | -0.013 | Balanced, <0.1 |
| Admission: Monday | 0.088 | 0.069 | Balanced, <0.1 |
| Admission: Tuesday | -0.055 | -0.037 | Balanced, <0.1 |
| Admission: Thursday | -0.010 | 0.035 | Balanced, <0.1 |
| Admission: Friday | 0.016 | 0.036 | Balanced, <0.1 |
| Admission: Saturday | -0.011 | -0.023 | Balanced, <0.1 |
| Admission: Sunday | 0.008 | -0.065 | Balanced, <0.1 |
| Heart rate1 | 0.125 | 0.043 | Balanced, <0.1 |
| Heart rate2 | 0.263 | -0.024 | Balanced, <0.1 |
| Respiratory rate | 0.326 | -0.044 | Balanced, <0.1 |
| SBP1 | -0.164 | 0.044 | Balanced, <0.1 |
| SBP2 | -0.176 | 0.094 | Balanced, <0.1 |
| SBP3 | -0.075 | -0.015 | Balanced, <0.1 |
| DBP1 | -0.230 | 0.077 | Balanced, <0.1 |
| DBP2 | -0.031 | -0.045 | Balanced, <0.1 |
| Temperature1 | -0.106 | 0.056 | Balanced, <0.1 |
| Temperature2 | 0.242 | -0.039 | Balanced, <0.1 |
| Saturation1 | -0.065 | 0.052 | Balanced, <0.1 |
| Saturation2 | -0.065 | -0.049 | Balanced, <0.1 |
| Use of oxygen supplement | 0.346 | 0.029 | Balanced, <0.1 |
| Oxygen flow rate | 0.431 | 0.055 | Balanced, <0.1 |
| AVPU:Alert | -0.254 | 0.020 | Balanced, <0.1 |
| AVPU:Pain | 0.121 | 0.085 | Balanced, <0.1 |
| AVPU:Unresponsive | 0.080 | 0.056 | Balanced, <0.1 |
| AVPU:Voice | 0.220 | -0.051 | Balanced, <0.1 |
| NEWS2 score | 0.536 | -0.048 | Balanced, <0.1 |
| Haemoglobin1 | -0.152 | 0.024 | Balanced, <0.1 |
| Haemoglobin2 | -0.018 | 0.062 | Balanced, <0.1 |
| MCV1 | -0.115 | -0.055 | Balanced, <0.1 |
| MCV2 | 0.088 | 0.057 | Balanced, <0.1 |
| Monocytes1 | -0.099 | 0.025 | Balanced, <0.1 |
| Monocytes2 | 0.165 | -0.021 | Balanced, <0.1 |
| Neutrophils | 0.379 | -0.077 | Balanced, <0.1 |
| Lymphocytes1 | -0.281 | 0.031 | Balanced, <0.1 |
| Lymphocytes2 | -0.049 | -0.001 | Balanced, <0.1 |
| Eosinophils1 | -0.297 | 0.069 | Balanced, <0.1 |
| Eosinophils2 | -0.082 | -0.088 | Balanced, <0.1 |
| Platelets1 | -0.001 | -0.081 | Balanced, <0.1 |
| Platelets2 | -0.184 | 0.028 | Balanced, <0.1 |
| Platelets3 | 0.140 | -0.009 | Balanced, <0.1 |
| Sodium1 | -0.178 | -0.046 | Balanced, <0.1 |
| Sodium2 | -0.001 | -0.030 | Balanced, <0.1 |
| Sodium3 | 0.104 | 0.047 | Balanced, <0.1 |
| Potassium1 | -0.015 | -0.060 | Balanced, <0.1 |
| Potassium2 | -0.104 | -0.023 | Balanced, <0.1 |
| Potassium3 | 0.109 | 0.035 | Balanced, <0.1 |
| Urea1 | 0.201 | 0.010 | Balanced, <0.1 |
| Urea2 | 0.133 | -0.066 | Balanced, <0.1 |
| Bilirubin | 0.122 | 0.004 | Balanced, <0.1 |
| ALT | 0.144 | 0.078 | Balanced, <0.1 |
| ALP1 | 0.121 | -0.021 | Balanced, <0.1 |
| ALP2 | 0.081 | -0.051 | Balanced, <0.1 |
| Albumin | -0.392 | 0.043 | Balanced, <0.1 |
| CRP1 | 0.184 | -0.086 | Balanced, <0.1 |
| CRP2 | 0.214 | 0.036 | Balanced, <0.1 |
| CRP3 | 0.105 | -0.001 | Balanced, <0.1 |
| PT1 | 0.253 | -0.030 | Balanced, <0.1 |
| PT2 | 0.253 | -0.096 | Balanced, <0.1 |
| PT3 | -0.202 | 0.037 | Balanced, <0.1 |
| PT4 | 0.161 | -0.039 | Balanced, <0.1 |
| APTT | 0.144 | -0.046 | Balanced, <0.1 |
| Lactate | 0.347 | 0.051 | Balanced, <0.1 |
| pH<7.25 | 0.191 | 0.117 | Not Balanced, >0.1 |
| CURB1 | 0.212 | -0.016 | Balanced, <0.1 |
| CURB2 | 0.184 | -0.081 | Balanced, <0.1 |
| SMART1 | 0.164 | -0.034 | Balanced, <0.1 |
| SMART2 | 0.310 | -0.012 | Balanced, <0.1 |
| PSI1 | 0.293 | -0.002 | Balanced, <0.1 |
| PSI2 | -0.162 | -0.006 | Balanced, <0.1 |
| PSI3 | 0.258 | -0.061 | Balanced, <0.1 |

**Table S3. Covariates’ means and standardised mean difference (SMD) between patients with baseline co-amoxiclav vs. amoxicillin post-propensity score matching.** SMD<0.1 indicates good covariate balance is achieved. IMD: index of multiple deprivation; UTI: urinary tract infection; SBP: systolic blood pressure; DBP: diastolic blood pressure; MCV: mean cell volume; ALT: alanine transferase; ALP: alkaline phosphatase; CRP: C-reactive protein; PT: prothrombin time; APTT: activated partial thromboplastin time; CURB: CURB-65 score; SMART: SMART-COP score; PSI: PSI/PORT score.

| **(A)    Method** |  | **Marginal OR** | **95%CI** | **p-value** |
| --- | --- | --- | --- | --- |
|  |  | **(Co-amoxiclav vs amoxicillin)** |  |  |
| **Complete cases (N=4136)** |  |  |  |  |
| **PS stratification** | p1 | 1.24 | 0.74-2.05 | 0.40 |
|  | p2 | 1.21 | 0.80-1.86 | 0.38 |
|  | p3 | 0.95 | 0.62-1.47 | 0.80 |
|  | p4 | 1.10 | 0.70-1.78 | 0.68 |
|  | p5 | 0.88 | 0.45-1.77 | 0.70 |
|  | pooled | 1.02 | 0.89-1.16 | 0.81 |
| **Doubly robust estimator** |  | 0.99 | 0.84-1.15 | 0.86 |
| **(B)    Method** |  | **Marginal OR** | **95%CI** | **p-value** |
|  |  | **(Co-amoxiclav vs amoxicillin)** |  |  |
| **Complete cases (N=2859)** |  |  |  |  |
| **Matching** | Univariable | 0.89 | 0.66-1.20 | 0.45 |
|  | Adjusted for variables with SMD>0.1 | 1.10 | 0.77-1.58 | 0.60 |
| **IPTW** | Univariable | 1.04 | 0.56-1.90 | 0.91 |
|  | Adjusted for variables with SMD>0.1 | 0.96 | 0.53-1.74 | 0.89 |
| **Multivariable logistic regression** |  | 1.24 | 0.86-1.79 | 0.26 |
| **(C) Method** |  | **Marginal OR** | **95%CI** | **p-value** |
|  |  | **(Co-amoxiclav vs amoxicillin)** |  |  |
| **Oral only (N=905)** | |  |  |  |
| **PS Matching** | Univariable | 1.08 | 0.55-2.13 | 0.82 |
|  | Adjusted for variables with SMD>0.1 | 1.09 | 0.60-2.00 | 0.78 |
| **IPTW** | Univariable | 0.97 | 0.54-1.75 | 0.91 |
|  | Adjusted for variables with SMD>0.1 | 0.96 | 0.53-1.75 | 0.90 |
| **Multivariable logistic regression** | | 0.84 | 0.38-1.80 | 0.67 |
| **Intravenous only (N=2681)** | |  |  |  |
| **PS Matching** | Univariable | 0.97 | 0.61-1.52 | 0.88 |
|  | Adjusted for variables with SMD>0.1 | 1.10 | 0.83-1.46 | 0.50 |
| **IPTW** | Univariable | 1.21 | 0.83-1.75 | 0.32 |
|  | Adjusted for variables with SMD>0.1 | 0.99 | 0.75-1.31 | 0.94 |
| **Multivariable logistic regression** | | 0.95 | 0.65-1.39 | 0.78 |

**Table S4. Average treatment effects (marginal odds ratios (ORs) and 95% confidence intervals (CIs)) of baseline co-amoxiclav vs amoxicillin on 30-day all-cause mortality in sensitivity analyses**. (A) Using propensity score stratification and doubly robust estimation in 4,136 admissions. (B) Using propensity score (PS) matching and inverse probability treatment weighting (IPTW) in 2,859 admissions with a confirmation of pneumonia from chest X-ray reports. (C) Using PS matching and IPTW in 905 admissions with only oral co-amoxiclav or amoxicillin, and 2681 admissions with only intravenous co-amoxiclav or amoxicillin.

|  | Raw difference | Standardized mean difference | Balance |
| --- | --- | --- | --- |
| distance | 1.191 | 0.058 | Balanced, <0.1 |
| Age | 0.045 | -0.035 | Balanced, <0.1 |
| Sex-Male | 0.115 | -0.009 | Balanced, <0.1 |
| Ethnicity-Non-white | 0.025 | 0.049 | Balanced, <0.1 |
| IMD | 0.054 | 0.009 | Balanced, <0.1 |
| Additional gentamicin | 0.469 | 0.077 | Balanced, <0.1 |
| Additional macrolide | 0.236 | -0.092 | Balanced, <0.1 |
| Additional doxycycline | -0.580 | -0.001 | Balanced, <0.1 |
| Consultant-Acute medicine | 0.095 | 0.000 | Balanced, <0.1 |
| Consultant-Emergency Medicine | -0.173 | -0.010 | Balanced, <0.1 |
| Consultant-Gerontology | -0.015 | -0.030 | Balanced, <0.1 |
| Consultant-Infectious disease | -0.062 | 0.040 | Balanced, <0.1 |
| Consultant-Other | 0.042 | 0.004 | Balanced, <0.1 |
| Charlson score1 | 0.170 | 0.011 | Balanced, <0.1 |
| Charlson score2 | -0.115 | 0.007 | Balanced, <0.1 |
| Frailty score1 | 0.215 | 0.040 | Balanced, <0.1 |
| Frailty score2 | 0.121 | 0.036 | Balanced, <0.1 |
| Frailty score3 | 0.072 | -0.034 | Balanced, <0.1 |
| Frailty score4 | -0.001 | 0.036 | Balanced, <0.1 |
| Previous length of stay1 | 0.179 | 0.013 | Balanced, <0.1 |
| Previous length of stay2 | 0.018 | 0.017 | Balanced, <0.1 |
| Admissions in previous year | -0.113 | -0.018 | Balanced, <0.1 |
| Palliative care | 0.201 | 0.001 | Balanced, <0.1 |
| UTI | 0.106 | 0.004 | Balanced, <0.1 |
| Immunosuppression | 0.146 | -0.049 | Balanced, <0.1 |
| Autoimmune diseases | 0.016 | -0.014 | Balanced, <0.1 |
| Admission Date | -0.186 | 0.039 | Balanced, <0.1 |
| Admission hour:11-15 | 0.133 | 0.030 | Balanced, <0.1 |
| Admission hour:0-8 | -0.053 | -0.005 | Balanced, <0.1 |
| Admission hour:8-11 | -0.047 | -0.003 | Balanced, <0.1 |
| Admission hour:15-24 | -0.022 | -0.027 | Balanced, <0.1 |
| Admission: Wednesday | 0.088 | 0.040 | Balanced, <0.1 |
| Admission: Monday | -0.055 | -0.025 | Balanced, <0.1 |
| Admission: Tuesday | -0.027 | -0.003 | Balanced, <0.1 |
| Admission: Thursday | -0.010 | 0.000 | Balanced, <0.1 |
| Admission: Friday | 0.016 | 0.043 | Balanced, <0.1 |
| Admission: Saturday | -0.011 | -0.019 | Balanced, <0.1 |
| Admission: Sunday | 0.008 | -0.033 | Balanced, <0.1 |
| Heart rate1 | 0.125 | 0.020 | Balanced, <0.1 |
| Heart rate2 | 0.263 | 0.048 | Balanced, <0.1 |
| Respiratory rate | 0.326 | 0.003 | Balanced, <0.1 |
| SBP1 | -0.164 | 0.012 | Balanced, <0.1 |
| SBP2 | -0.176 | -0.006 | Balanced, <0.1 |
| SBP3 | -0.075 | 0.015 | Balanced, <0.1 |
| DBP1 | -0.230 | 0.015 | Balanced, <0.1 |
| DBP2 | -0.031 | 0.007 | Balanced, <0.1 |
| Temperature1 | -0.106 | 0.003 | Balanced, <0.1 |
| Temperature2 | 0.242 | -0.004 | Balanced, <0.1 |
| Saturation1 | -0.065 | -0.002 | Balanced, <0.1 |
| Saturation2 | -0.065 | 0.027 | Balanced, <0.1 |
| Use of oxygen supplement | 0.346 | 0.063 | Balanced, <0.1 |
| Oxygen flow rate | 0.431 | 0.100 | Balanced, <0.1 |
| AVPU:Alert | -0.254 | -0.070 | Balanced, <0.1 |
| AVPU:Pain | 0.121 | 0.082 | Balanced, <0.1 |
| AVPU:Unresponsive | 0.080 | 0.054 | Balanced, <0.1 |
| AVPU:Voice | 0.220 | 0.042 | Balanced, <0.1 |
| NEWS2 score | 0.536 | 0.044 | Balanced, <0.1 |
| Haemoglobin1 | -0.152 | -0.009 | Balanced, <0.1 |
| Haemoglobin2 | -0.018 | 0.023 | Balanced, <0.1 |
| MCV1 | -0.115 | -0.033 | Balanced, <0.1 |
| MCV2 | 0.088 | 0.047 | Balanced, <0.1 |
| Monocytes1 | -0.099 | -0.009 | Balanced, <0.1 |
| Monocytes2 | 0.165 | -0.018 | Balanced, <0.1 |
| Neutrophils | 0.379 | 0.001 | Balanced, <0.1 |
| Lymphocytes1 | -0.281 | -0.002 | Balanced, <0.1 |
| Lymphocytes2 | -0.049 | 0.000 | Balanced, <0.1 |
| Eosinophils1 | -0.297 | -0.002 | Balanced, <0.1 |
| Eosinophils2 | -0.082 | -0.039 | Balanced, <0.1 |
| Platelets1 | -0.001 | -0.016 | Balanced, <0.1 |
| Platelets2 | -0.184 | -0.026 | Balanced, <0.1 |
| Platelets3 | 0.140 | 0.027 | Balanced, <0.1 |
| Sodium1 | -0.178 | -0.015 | Balanced, <0.1 |
| Sodium2 | -0.001 | -0.021 | Balanced, <0.1 |
| Sodium3 | 0.104 | 0.043 | Balanced, <0.1 |
| Potassium1 | -0.015 | -0.046 | Balanced, <0.1 |
| Potassium2 | -0.104 | -0.026 | Balanced, <0.1 |
| Potassium3 | 0.109 | 0.029 | Balanced, <0.1 |
| Urea1 | 0.201 | 0.017 | Balanced, <0.1 |
| Urea2 | 0.133 | 0.021 | Balanced, <0.1 |
| Bilirubin | 0.122 | -0.001 | Balanced, <0.1 |
| ALT | 0.144 | 0.058 | Balanced, <0.1 |
| ALP1 | 0.121 | 0.026 | Balanced, <0.1 |
| ALP2 | 0.081 | -0.005 | Balanced, <0.1 |
| Albumin | -0.392 | -0.018 | Balanced, <0.1 |
| CRP1 | 0.184 | -0.039 | Balanced, <0.1 |
| CRP2 | 0.214 | 0.008 | Balanced, <0.1 |
| CRP3 | 0.105 | 0.023 | Balanced, <0.1 |
| PT1 | 0.253 | 0.005 | Balanced, <0.1 |
| PT2 | 0.253 | -0.036 | Balanced, <0.1 |
| PT3 | -0.202 | 0.018 | Balanced, <0.1 |
| PT4 | 0.161 | -0.044 | Balanced, <0.1 |
| APTT | 0.144 | -0.028 | Balanced, <0.1 |
| Lactate | 0.347 | 0.078 | Balanced, <0.1 |
| pH<7.25 | 0.191 | 0.106 | Not Balanced, >0.1 |
| CURB1 | 0.212 | -0.003 | Balanced, <0.1 |
| CURB2 | 0.184 | 0.016 | Balanced, <0.1 |
| SMART1 | 0.164 | 0.019 | Balanced, <0.1 |
| SMART2 | 0.310 | 0.054 | Balanced, <0.1 |
| PSI1 | 0.293 | 0.034 | Balanced, <0.1 |
| PSI2 | -0.162 | -0.038 | Balanced, <0.1 |
| PSI3 | 0.258 | 0.036 | Balanced, <0.1 |

**Table S5. Covariates’ means and standardised mean difference (SMD) between patients with baseline co-amoxiclav vs. amoxicillin post-inverse probability treatment weighting (IPTW).** SMD<0.1 indicates good covariate balance is achieved. Extreme weights were truncated at 99.9% centile. IMD: index of multiple deprivation; UTI: urinary tract infection; SBP: systolic blood pressure; DBP: diastolic blood pressure; MCV: mean cell volume; ALT: alanine transferase; ALP: alkaline phosphatase; CRP: C-reactive protein; PT: prothrombin time; APTT: activated partial thromboplastin time; CURB: CURB-65 score; SMART: SMART-COP score; PSI: PSI/PORT score.

| Covariate | Proportion of missingness (%) |
| --- | --- |
| Lactate | 21.3 |
| APTT | 15.7 |
| PT | 15.1 |
| ALT | 8.0 |
| Bilirubin | 8.0 |
| ALP | 7.6 |
| Albumin | 7.3 |
| CRP | 6.8 |
| Oxygen Saturation | 5.5 |
| Oxygen flowrate | 4.8 |
| Use of oxygen supplement | 4.8 |
| Heart rate | 4.8 |
| Systolic blood pressure | 4.8 |
| Diastolic blood pressure | 4.8 |
| Respiratory rate | 4.8 |
| Temperature | 4.8 |
| Potassium | 4.5 |
| Eosinophils | 2.8 |
| Lymphocytes | 2.8 |
| Monocytes | 2.8 |
| Neutrophils | 2.8 |
| Platelets | 2.5 |
| MCV | 2.5 |
| Haemoglobin | 2.4 |
| Urea | 2.4 |
| Sodium | 2.4 |
| IMD score | 1.1 |

**Table S6. Proportion of missing covariates.** APTT: activated partial thromboplastin time; PT: prothrombin time; ALT: alanine transferase; ALP: alkaline phosphatase; CRP: C-reactive protein; MCV: mean cell volume; IMD: index of multiple deprivation.

|  | Raw (min) | Raw (mean) | Raw (max) | SMD (min) | SMD (mean) | SMD (max) |
| --- | --- | --- | --- | --- | --- | --- |
| distance | 1.143 | 1.152 | 1.158 | 0.000 | 0.000 | 0.000 |
| Age | 0.150 | 0.150 | 0.150 | -0.077 | -0.031 | 0.000 |
| Sex-Male | 0.088 | 0.088 | 0.088 | -0.042 | -0.011 | 0.019 |
| Ethnicity-Non-white | -0.003 | -0.003 | -0.003 | -0.015 | 0.023 | 0.053 |
| IMD | 0.014 | 0.018 | 0.021 | -0.033 | 0.006 | 0.039 |
| Additional gentamicin | 0.457 | 0.457 | 0.457 | -0.074 | -0.030 | 0.013 |
| Additional macrolide | 0.269 | 0.269 | 0.269 | -0.149 | -0.119 | -0.081 |
| Additional doxycycline | -0.517 | -0.517 | -0.517 | -0.027 | -0.007 | 0.018 |
| Consultant-Acute medicine | 0.030 | 0.030 | 0.030 | -0.049 | -0.015 | 0.025 |
| Consultant-Emergency Medicine | -0.188 | -0.188 | -0.188 | -0.035 | -0.001 | 0.014 |
| Consultant-Gerontology | 0.006 | 0.006 | 0.006 | -0.021 | -0.004 | 0.016 |
| Consultant-Infectious disease | -0.028 | -0.028 | -0.028 | -0.005 | 0.029 | 0.062 |
| Consultant-Other | 0.098 | 0.098 | 0.098 | -0.042 | 0.002 | 0.033 |
| Charlson score1 | 0.236 | 0.236 | 0.236 | -0.044 | -0.017 | 0.017 |
| Charlson score2 | -0.138 | -0.138 | -0.138 | -0.026 | 0.014 | 0.048 |
| Frailty score1 | 0.305 | 0.305 | 0.305 | -0.025 | 0.019 | 0.051 |
| Frailty score2 | 0.193 | 0.193 | 0.193 | -0.048 | 0.004 | 0.037 |
| Frailty score3 | 0.063 | 0.063 | 0.063 | -0.041 | -0.010 | 0.038 |
| Frailty score4 | 0.018 | 0.018 | 0.018 | -0.046 | 0.009 | 0.040 |
| Previous length of stay1 | 0.234 | 0.234 | 0.234 | -0.021 | 0.012 | 0.063 |
| Previous length of stay2 | 0.050 | 0.050 | 0.050 | -0.019 | 0.024 | 0.061 |
| Admissions in previous year | -0.173 | -0.173 | -0.173 | -0.063 | -0.020 | 0.013 |
| Palliative care | 0.221 | 0.221 | 0.221 | -0.097 | -0.040 | 0.007 |
| UTI | 0.151 | 0.151 | 0.151 | -0.049 | 0.014 | 0.065 |
| Immunosuppression | 0.149 | 0.149 | 0.149 | -0.058 | -0.017 | 0.015 |
| Autoimmune diseases | 0.047 | 0.047 | 0.047 | -0.088 | -0.033 | 0.017 |
| Admission Date | -0.250 | -0.250 | -0.250 | 0.039 | 0.070 | 0.094 |
| Admission hour:0-8 | 0.146 | 0.146 | 0.146 | -0.063 | -0.026 | 0.011 |
| Admission hour:8-11 | -0.099 | -0.099 | -0.099 | -0.033 | 0.000 | 0.031 |
| Admission hour:11-15 | -0.097 | -0.097 | -0.097 | -0.068 | -0.006 | 0.028 |
| Admission hour:15-24 | 0.042 | 0.042 | 0.042 | -0.005 | 0.024 | 0.074 |
| Admission: Wednesday | 0.050 | 0.050 | 0.050 | -0.045 | 0.015 | 0.058 |
| Admission: Monday | 0.001 | 0.001 | 0.001 | -0.071 | -0.030 | 0.003 |
| Admission: Tuesday | -0.020 | -0.020 | -0.020 | -0.049 | -0.004 | 0.033 |
| Admission: Thursday | -0.002 | -0.002 | -0.002 | -0.020 | 0.023 | 0.065 |
| Admission: Friday | -0.013 | -0.013 | -0.013 | -0.016 | 0.030 | 0.067 |
| Admission: Saturday | -0.001 | -0.001 | -0.001 | -0.051 | -0.014 | 0.025 |
| Admission: Sunday | -0.012 | -0.012 | -0.012 | -0.064 | -0.018 | 0.026 |
| Heart rate1 | 0.089 | 0.098 | 0.108 | 0.018 | 0.046 | 0.082 |
| Heart rate2 | 0.230 | 0.241 | 0.254 | -0.022 | 0.029 | 0.066 |
| Respiratory rate | 0.306 | 0.316 | 0.325 | -0.075 | -0.021 | 0.040 |
| SBP1 | -0.172 | -0.158 | -0.150 | 0.007 | 0.026 | 0.062 |
| SBP2 | -0.204 | -0.196 | -0.187 | -0.035 | 0.019 | 0.055 |
| SBP3 | -0.055 | -0.045 | -0.039 | -0.010 | 0.018 | 0.053 |
| DBP1 | -0.268 | -0.260 | -0.247 | 0.006 | 0.059 | 0.111 |
| DBP2 | -0.022 | -0.011 | -0.004 | -0.033 | 0.004 | 0.050 |
| Temperature1 | -0.132 | -0.121 | -0.114 | -0.031 | 0.009 | 0.038 |
| Temperature2 | 0.208 | 0.222 | 0.236 | -0.051 | 0.017 | 0.057 |
| Saturation1 | -0.108 | -0.100 | -0.092 | -0.037 | 0.005 | 0.053 |
| Saturation2 | -0.077 | -0.067 | -0.054 | -0.055 | -0.009 | 0.035 |
| Use of oxygen supplement | 0.359 | 0.371 | 0.382 | -0.031 | -0.003 | 0.077 |
| Oxygen flow rate | 0.408 | 0.424 | 0.433 | 0.000 | 0.033 | 0.082 |
| AVPU:Alert | -0.277 | -0.277 | -0.277 | -0.046 | 0.008 | 0.049 |
| AVPU:Pain | 0.124 | 0.124 | 0.124 | -0.130 | -0.045 | 0.061 |
| AVPU:Unresponsive | 0.082 | 0.082 | 0.082 | 0.055 | 0.055 | 0.055 |
| AVPU:Voice | 0.239 | 0.239 | 0.239 | -0.070 | -0.005 | 0.036 |
| NEWS2 score | 0.560 | 0.574 | 0.583 | -0.035 | -0.012 | 0.020 |
| Haemoglobin1 | -0.175 | -0.168 | -0.161 | -0.051 | -0.010 | 0.041 |
| Haemoglobin2 | -0.088 | -0.080 | -0.075 | -0.016 | 0.024 | 0.058 |
| MCV1 | -0.102 | -0.090 | -0.082 | -0.072 | -0.025 | 0.000 |
| MCV2 | 0.111 | 0.121 | 0.129 | -0.027 | 0.015 | 0.047 |
| Monocytes1 | -0.092 | -0.085 | -0.080 | -0.041 | -0.007 | 0.048 |
| Monocytes2 | 0.161 | 0.171 | 0.180 | -0.080 | -0.024 | 0.017 |
| Neutrophils | 0.370 | 0.377 | 0.384 | -0.068 | -0.032 | 0.000 |
| Lymphocytes1 | -0.287 | -0.280 | -0.273 | -0.001 | 0.027 | 0.065 |
| Lymphocytes2 | -0.063 | -0.056 | -0.051 | -0.053 | -0.013 | 0.020 |
| Eosinophils1 | -0.301 | -0.296 | -0.291 | -0.039 | -0.003 | 0.016 |
| Eosinophils2 | -0.065 | -0.055 | -0.046 | -0.067 | -0.028 | 0.006 |
| Platelets1 | -0.009 | -0.002 | 0.002 | -0.073 | -0.037 | 0.007 |
| Platelets2 | -0.156 | -0.149 | -0.140 | -0.028 | 0.037 | 0.063 |
| Platelets3 | 0.109 | 0.115 | 0.123 | -0.041 | -0.013 | 0.019 |
| Sodium1 | -0.251 | -0.244 | -0.239 | -0.057 | -0.024 | 0.008 |
| Sodium2 | 0.008 | 0.015 | 0.021 | -0.044 | 0.002 | 0.051 |
| Sodium3 | 0.063 | 0.070 | 0.077 | 0.000 | 0.035 | 0.081 |
| Potassium1 | -0.014 | 0.000 | 0.008 | -0.073 | -0.043 | -0.006 |
| Potassium2 | -0.115 | -0.108 | -0.099 | -0.029 | -0.004 | 0.019 |
| Potassium3 | 0.138 | 0.144 | 0.150 | -0.071 | -0.025 | 0.019 |
| Urea1 | 0.246 | 0.252 | 0.258 | -0.048 | -0.017 | 0.007 |
| Urea2 | 0.149 | 0.154 | 0.163 | -0.075 | -0.024 | 0.014 |
| Bilirubin | 0.094 | 0.106 | 0.114 | -0.071 | -0.023 | 0.022 |
| ALT | 0.083 | 0.092 | 0.105 | -0.008 | 0.031 | 0.060 |
| ALP1 | 0.160 | 0.170 | 0.178 | -0.023 | 0.015 | 0.058 |
| ALP2 | 0.063 | 0.076 | 0.087 | -0.045 | -0.014 | 0.024 |
| Albumin | -0.477 | -0.463 | -0.452 | -0.042 | -0.015 | 0.042 |
| CRP1 | 0.180 | 0.189 | 0.199 | -0.100 | -0.064 | -0.014 |
| CRP2 | 0.175 | 0.186 | 0.194 | -0.027 | 0.006 | 0.071 |
| CRP3 | 0.125 | 0.135 | 0.146 | -0.050 | 0.016 | 0.055 |
| PT1 | 0.237 | 0.249 | 0.263 | -0.037 | 0.008 | 0.052 |
| PT2 | 0.246 | 0.260 | 0.269 | -0.091 | -0.055 | -0.012 |
| PT3 | -0.193 | -0.181 | -0.164 | -0.015 | 0.017 | 0.050 |
| PT4 | 0.140 | 0.160 | 0.178 | -0.155 | -0.052 | -0.016 |
| APTT | 0.138 | 0.158 | 0.175 | -0.117 | -0.051 | 0.006 |
| Lactate | 0.296 | 0.314 | 0.330 | -0.013 | 0.032 | 0.099 |
| pH<7.25 | 0.186 | 0.186 | 0.186 | 0.021 | 0.071 | 0.105 |
| CURB1 | 0.300 | 0.304 | 0.308 | -0.060 | -0.034 | -0.014 |
| CURB2 | 0.111 | 0.116 | 0.122 | -0.093 | -0.027 | 0.026 |
| SMART1 | 0.227 | 0.241 | 0.256 | -0.057 | 0.000 | 0.043 |
| SMART2 | 0.277 | 0.294 | 0.305 | -0.073 | 0.004 | 0.037 |
| PSI1 | 0.343 | 0.350 | 0.354 | -0.041 | -0.012 | 0.026 |
| PSI2 | -0.078 | -0.070 | -0.054 | -0.038 | -0.003 | 0.029 |
| PSI3 | 0.229 | 0.240 | 0.250 | -0.064 | -0.022 | 0.025 |

**Table S7. Covariates’ raw and standardised mean difference (SMD) between patients with baseline co-amoxiclav vs. amoxicillin post-PS matching in imputed datasets.** Minimum, mean, and maximum differences in 25 imputed datasets were shown. SMD<0.1 indicates good covariate balance is achieved. IMD: index of multiple deprivation; UTI: urinary tract infection; SBP: systolic blood pressure; DBP: diastolic blood pressure; MCV: mean cell volume; ALT: alanine transferase; ALP: alkaline phosphatase; CRP: C-reactive protein; PT: prothrombin time; APTT: activated partial thromboplastin time; CURB: CURB-65 score; SMART: SMART-COP score; PSI: PSI/PORT score.

|  | Raw (min) | Raw (mean) | Raw (max) | SMD (min) | SMD (mean) | SMD (max) |
| --- | --- | --- | --- | --- | --- | --- |
| distance | 1.143 | 1.152 | 1.158 | -0.048 | -0.012 | 0.006 |
| Age | 0.150 | 0.150 | 0.150 | -0.037 | -0.027 | -0.018 |
| Sex-Male | 0.088 | 0.088 | 0.088 | -0.014 | 0.000 | 0.014 |
| Ethnicity-Non-white | -0.003 | -0.003 | -0.003 | 0.015 | 0.022 | 0.030 |
| IMD | 0.014 | 0.018 | 0.021 | -0.010 | 0.004 | 0.012 |
| Additional gentamicin | 0.457 | 0.457 | 0.457 | -0.115 | -0.041 | -0.007 |
| Additional macrolide | 0.269 | 0.269 | 0.269 | -0.152 | -0.115 | -0.099 |
| Additional doxycycline | -0.517 | -0.517 | -0.517 | 0.009 | 0.015 | 0.023 |
| Consultant-Acute medicine | 0.030 | 0.030 | 0.030 | -0.047 | -0.018 | -0.007 |
| Consultant-Emergency Medicine | -0.188 | -0.188 | -0.188 | -0.007 | -0.003 | 0.001 |
| Consultant-Gerontology | 0.006 | 0.006 | 0.006 | -0.013 | -0.005 | 0.010 |
| Consultant-Infectious disease | -0.028 | -0.028 | -0.028 | 0.025 | 0.033 | 0.041 |
| Consultant-Other | 0.098 | 0.098 | 0.098 | -0.006 | 0.005 | 0.020 |
| Charlson score1 | 0.236 | 0.236 | 0.236 | -0.031 | -0.018 | -0.009 |
| Charlson score2 | -0.138 | -0.138 | -0.138 | 0.003 | 0.011 | 0.021 |
| Frailty score1 | 0.305 | 0.305 | 0.305 | 0.012 | 0.021 | 0.030 |
| Frailty score2 | 0.193 | 0.193 | 0.193 | -0.008 | 0.015 | 0.024 |
| Frailty score3 | 0.063 | 0.063 | 0.063 | -0.021 | -0.015 | -0.008 |
| Frailty score4 | 0.018 | 0.018 | 0.018 | 0.007 | 0.016 | 0.022 |
| Previous length of stay1 | 0.234 | 0.234 | 0.234 | 0.009 | 0.018 | 0.027 |
| Previous length of stay2 | 0.063 | 0.063 | 0.063 | 0.009 | 0.018 | 0.023 |
| Admissions in previous year | -0.173 | -0.173 | -0.173 | -0.034 | -0.025 | -0.017 |
| Palliative care | 0.221 | 0.221 | 0.221 | -0.082 | -0.037 | -0.018 |
| UTI | 0.151 | 0.151 | 0.151 | 0.009 | 0.019 | 0.031 |
| Immunosuppression | 0.149 | 0.149 | 0.149 | -0.038 | -0.026 | -0.010 |
| Autoimmune diseases | 0.047 | 0.047 | 0.047 | -0.037 | -0.028 | -0.019 |
| Admission Date | -0.250 | -0.250 | -0.250 | 0.062 | 0.080 | 0.098 |
| Admission hour:11-15 | 0.146 | 0.146 | 0.146 | -0.048 | -0.025 | -0.011 |
| Admission hour:0-8 | -0.097 | -0.097 | -0.097 | -0.014 | 0.003 | 0.012 |
| Admission hour:8-11 | 0.042 | 0.042 | 0.042 | 0.002 | 0.013 | 0.041 |
| Admission hour:15-24 | -0.099 | -0.099 | -0.099 | -0.004 | 0.004 | 0.009 |
| Admission: Wednesday | 0.050 | 0.050 | 0.050 | -0.005 | 0.009 | 0.019 |
| Admission: Monday | 0.001 | 0.001 | 0.001 | -0.029 | -0.019 | -0.010 |
| Admission: Tuesday | -0.020 | -0.020 | -0.020 | -0.019 | -0.008 | 0.000 |
| Admission: Thursday | -0.002 | -0.002 | -0.002 | 0.015 | 0.025 | 0.038 |
| Admission: Friday | -0.013 | -0.013 | -0.013 | 0.029 | 0.036 | 0.046 |
| Admission: Saturday | -0.001 | -0.001 | -0.001 | -0.043 | -0.027 | -0.016 |
| Admission: Sunday | -0.012 | -0.012 | -0.012 | -0.032 | -0.015 | 0.002 |
| Heart rate1 | 0.089 | 0.098 | 0.108 | 0.025 | 0.035 | 0.053 |
| Heart rate2 | 0.230 | 0.241 | 0.254 | -0.013 | 0.025 | 0.038 |
| Respiratory rate | 0.306 | 0.316 | 0.325 | -0.071 | -0.030 | -0.010 |
| SBP1 | -0.172 | -0.158 | -0.150 | 0.027 | 0.039 | 0.056 |
| SBP2 | -0.204 | -0.196 | -0.187 | -0.009 | 0.014 | 0.065 |
| SBP3 | -0.055 | -0.045 | -0.039 | 0.003 | 0.020 | 0.028 |
| DBP1 | -0.268 | -0.260 | -0.247 | 0.027 | 0.049 | 0.080 |
| DBP2 | -0.022 | -0.011 | -0.004 | 0.001 | 0.012 | 0.021 |
| Temperature1 | -0.132 | -0.121 | -0.114 | -0.009 | 0.008 | 0.035 |
| Temperature2 | 0.208 | 0.222 | 0.236 | -0.005 | 0.020 | 0.034 |
| Saturation1 | -0.108 | -0.100 | -0.092 | -0.004 | 0.015 | 0.063 |
| Saturation2 | -0.077 | -0.067 | -0.054 | -0.030 | -0.006 | 0.009 |
| Use of oxygen supplement | 0.359 | 0.371 | 0.382 | -0.033 | -0.001 | 0.023 |
| Oxygen flow rate | 0.408 | 0.424 | 0.433 | -0.081 | 0.023 | 0.056 |
| AVPU:Alert | -0.277 | -0.277 | -0.277 | -0.030 | -0.018 | 0.007 |
| AVPU:Pain | 0.124 | 0.124 | 0.124 | -0.078 | -0.035 | -0.011 |
| AVPU:Unresponsive | 0.082 | 0.082 | 0.082 | 0.053 | 0.053 | 0.053 |
| AVPU:Voice | 0.239 | 0.239 | 0.239 | -0.006 | 0.019 | 0.027 |
| NEWS2 score | 0.560 | 0.574 | 0.583 | -0.056 | -0.011 | 0.019 |
| Haemoglobin1 | -0.175 | -0.168 | -0.161 | -0.011 | -0.003 | 0.015 |
| Haemoglobin2 | -0.088 | -0.080 | -0.075 | 0.015 | 0.025 | 0.036 |
| MCV1 | -0.102 | -0.090 | -0.082 | -0.027 | -0.018 | -0.006 |
| MCV2 | 0.111 | 0.121 | 0.129 | 0.006 | 0.022 | 0.035 |
| Monocytes1 | -0.092 | -0.085 | -0.080 | -0.029 | -0.019 | -0.002 |
| Monocytes2 | 0.161 | 0.171 | 0.180 | -0.025 | -0.013 | 0.000 |
| Neutrophils | 0.370 | 0.377 | 0.384 | -0.071 | -0.047 | -0.036 |
| Lymphocytes1 | -0.287 | -0.280 | -0.273 | 0.005 | 0.014 | 0.031 |
| Lymphocytes2 | -0.063 | -0.056 | -0.051 | -0.029 | -0.012 | 0.001 |
| Eosinophils1 | -0.301 | -0.296 | -0.291 | -0.011 | -0.001 | 0.011 |
| Eosinophils2 | -0.065 | -0.055 | -0.046 | -0.061 | -0.034 | -0.016 |
| Platelets1 | -0.009 | -0.002 | 0.002 | -0.065 | -0.050 | -0.039 |
| Platelets2 | -0.156 | -0.149 | -0.140 | 0.018 | 0.032 | 0.050 |
| Platelets3 | 0.109 | 0.115 | 0.123 | -0.031 | -0.018 | -0.007 |
| Sodium1 | -0.251 | -0.244 | -0.239 | -0.029 | -0.018 | -0.007 |
| Sodium2 | 0.008 | 0.015 | 0.021 | -0.014 | -0.003 | 0.006 |
| Sodium3 | 0.063 | 0.070 | 0.077 | 0.024 | 0.032 | 0.045 |
| Potassium1 | -0.014 | 0.000 | 0.008 | -0.069 | -0.040 | -0.021 |
| Potassium2 | -0.115 | -0.108 | -0.099 | -0.009 | -0.001 | 0.015 |
| Potassium3 | 0.138 | 0.144 | 0.150 | -0.056 | -0.026 | -0.012 |
| Urea1 | 0.246 | 0.252 | 0.258 | -0.022 | -0.011 | 0.008 |
| Urea2 | 0.149 | 0.154 | 0.163 | -0.044 | -0.020 | -0.007 |
| Bilirubin | 0.094 | 0.106 | 0.114 | -0.062 | -0.015 | 0.002 |
| ALT | 0.083 | 0.092 | 0.105 | -0.007 | 0.035 | 0.055 |
| ALP1 | 0.160 | 0.170 | 0.178 | -0.006 | 0.012 | 0.031 |
| ALP2 | 0.063 | 0.076 | 0.087 | -0.039 | -0.018 | -0.004 |
| Albumin | -0.477 | -0.463 | -0.452 | -0.017 | -0.004 | 0.014 |
| CRP1 | 0.180 | 0.189 | 0.199 | -0.086 | -0.057 | -0.037 |
| CRP2 | 0.175 | 0.186 | 0.194 | -0.017 | 0.002 | 0.016 |
| CRP3 | 0.125 | 0.135 | 0.146 | -0.026 | 0.013 | 0.031 |
| PT1 | 0.237 | 0.249 | 0.263 | -0.018 | -0.004 | 0.020 |
| PT2 | 0.246 | 0.260 | 0.269 | -0.110 | -0.057 | -0.026 |
| PT3 | -0.193 | -0.181 | -0.164 | 0.005 | 0.022 | 0.036 |
| PT4 | 0.140 | 0.160 | 0.178 | -0.102 | -0.055 | -0.037 |
| APTT | 0.138 | 0.158 | 0.175 | -0.096 | -0.053 | -0.026 |
| Lactate | 0.296 | 0.314 | 0.330 | -0.018 | 0.019 | 0.039 |
| pH<7.25 | 0.186 | 0.186 | 0.186 | 0.056 | 0.069 | 0.080 |
| CURB1 | 0.300 | 0.304 | 0.308 | -0.039 | -0.030 | -0.018 |
| CURB2 | 0.111 | 0.116 | 0.122 | -0.044 | -0.023 | -0.002 |
| SMART1 | 0.227 | 0.241 | 0.256 | -0.014 | 0.004 | 0.019 |
| SMART2 | 0.277 | 0.294 | 0.305 | -0.102 | -0.010 | 0.026 |
| PSI1 | 0.343 | 0.350 | 0.354 | -0.038 | -0.013 | -0.001 |
| PSI2 | -0.078 | -0.070 | -0.054 | -0.010 | 0.003 | 0.029 |
| PSI3 | 0.229 | 0.240 | 0.250 | -0.091 | -0.023 | -0.002 |

**Table S8. Covariates’ raw and standardised mean difference (SMD) between patients with baseline co-amoxiclav vs. amoxicillin post-inverse probability treatment weighting in imputed datasets.** Minimum, mean, and maximum differences in 25 imputed datasets were shown. SMD<0.1 indicates good covariate balance is achieved. Extreme weights were truncated at 99.9% centile. IMD: index of multiple deprivation; UTI: urinary tract infection; SBP: systolic blood pressure; DBP: diastolic blood pressure; MCV: mean cell volume; ALT: alanine transferase; ALP: alkaline phosphatase; CRP: C-reactive protein; PT: prothrombin time; APTT: activated partial thromboplastin time; CURB: CURB-65 score; SMART: SMART-COP score; PSI: PSI/PORT score.

|  | OR | Lower CI | Upper CI | p-value |
| --- | --- | --- | --- | --- |
| Treatment (Co-amoxiclav vs Amoxicillin) | 1.08 | 0.84 | 1.41 | 0.54 |
| Age (per 10 years older) | 1.46 | 1.27 | 1.70 | <0.001 |
| Sex (Male vs Female) | 0.82 | 0.66 | 1.04 | 0.10 |
| Ethnicity (Non-white vs White) | 0.94 | 0.46 | 1.82 | 0.86 |
| IMD score (per 10 higher) | 0.96 | 0.86 | 1.08 | 0.54 |
| Additional gentamicin | 0.98 | 0.71 | 1.34 | 0.89 |
| Additional macrolide | 1.13 | 0.89 | 1.44 | 0.33 |
| Additional doxycycline | 1.00 | 0.72 | 1.38 | 0.99 |
| Consultant specialty (Emergency Medicine vs Acute Medicine) | 0.97 | 0.45 | 1.93 | 0.93 |
| Consultant specialty (Gerontology vs Acute Medicine) | 1.20 | 0.94 | 1.54 | 0.15 |
| Consultant specialty (Infectious disease vs Acute Medicine) | 0.82 | 0.58 | 1.14 | 0.24 |
| Consultant specialty (Other vs Acute Medicine) | 1.48 | 1.09 | 1.99 | 0.01 |
| Hospital admission in previous year (Yes vs No) | 0.98 | 0.75 | 1.29 | 0.89 |
| Palliative care (Yes vs No) | 12.36 | 9.20 | 16.71 | <0.001 |
| UTI (Yes vs No) | 0.67 | 0.47 | 0.96 | 0.03 |
| Immunosuppression (Yes vs No) | 1.29 | 0.89 | 1.84 | 0.17 |
| Autoimmune diseases (Yes vs No) | 1.02 | 0.75 | 1.38 | 0.884 |
| Changes over calendar time | 1.00 | 1.00 | 1.00 | 0.15 |
| Admission hour (0-8 vs 11-15) | 0.69 | 0.49 | 0.96 | 0.03 |
| Admission hour (8-11 vs 11-15) | 1.01 | 0.68 | 1.49 | 0.97 |
| Admission hour (15-24 vs 11-15) | 0.95 | 0.73 | 1.24 | 0.70 |
| Admission date (Monday vs Wednesday) | 1.23 | 0.84 | 1.81 | 0.29 |
| Admission date (Tuesday vs Wednesday) | 1.22 | 0.84 | 1.77 | 0.30 |
| Admission date (Thursday vs Wednesday) | 1.60 | 1.11 | 2.31 | 0.01 |
| Admission date (Friday vs Wednesday) | 0.80 | 0.53 | 1.20 | 0.29 |
| Admission date (Saturday vs Wednesday) | 1.20 | 0.82 | 1.76 | 0.34 |
| Admission date (Sunday vs Wednesday) | 1.35 | 0.93 | 1.97 | 0.12 |
| Oxygen flow rate | 1.04 | 1.01 | 1.08 | 0.02 |
| Use oxygen supplement (Yes vs No) | 1.40 | 0.93 | 2.10 | 0.10 |
| AVPU (pain vs alert) | 4.06 | 1.23 | 13.59 | 0.02 |
| AVPU (voice vs alert) | 2.72 | 1.75 | 4.23 | <0.001 |
| AVPU (unresponsive vs alert) | 8.29 | 0.75 | 245.73 | 0.14 |
| NEWS2 score | 0.95 | 0.87 | 1.05 | 0.32 |
| Respiratory rate | 1.05 | 1.02 | 1.09 | 0.002 |
| APTT | 1.02 | 1.00 | 1.05 | 0.11 |
| Albumin | 0.91 | 0.88 | 0.94 | <0.001 |
| Neutrophils | 0.97 | 0.95 | 1.00 | 0.05 |
| ALT (per 10 higher) | 1.01 | 0.96 | 1.05 | 0.82 |
| Bilirubin (per 10 higher) | 1.03 | 0.89 | 1.19 | 0.71 |
| Lactate | 1.23 | 1.10 | 1.38 | <0.001 |
| pH<7.25 (Yes vs No) | 1.05 | 0.60 | 1.82 | 0.86 |

**Table S9. Associations (odds ratios (ORs) and 95% confidence intervals (95%CIs)) between baseline characteristics and 30-day all-cause mortality.** The 95% CIs are calculated as estimates ± 1.96 × standard error of the estimates. Non-linear effects are shown in **Figure S5, S6**. UTI: urinary tract infection; APTT: activated partial thromboplastin time.
